# Iron and its import systems enhance copper accumulation in *Streptococcus pneumoniae*

**DOI:** 10.1128/msphere.00165-26

**Published:** 2026-06-10

**Authors:** Yamil Sanchez-Rosario, Meredythe Durckel, Rithy Meas, Mahi Rohilla, Sakshi Parate, S. G. M. S. D. Senanayaka, Juan Angel Cota Ibarra, Igor H. Wierzbicki, David J. Gonzalez, Michael D. L. Johnson

**Affiliations:** 1 Department of Immunobiology, University of Arizona College of Medicine 12216, Tucson, Arizona, USA; 2 Department of Chemistry and Biochemistry, University of Arizona College of Medicine 12216, Tucson, Arizona, USA; 3 Department of Pharmacology, University of California, San Diego, California, USA; 4 Skaggs School of Pharmacy and Pharmaceutical Sciences, University of California, San Diego, California, USA; 5 Valley Fever Center for Excellence, University of Arizona College of Medicine 12216, Tucson, Arizona, USA; 6 BIO5 Institute, University of Arizona College of Medicine 124486, Tucson, Arizona, USA; 7 Asthma and Airway Disease Research Center, University of Arizona College of Medicine 12216, Tucson, Arizona, USA; The University of Iowa, Iowa City, lowa, USA

**Keywords:** copper, iron, substrate-binding protein, Pia, Piu, *Streptococcus pneumoniae*

## Abstract

Metal ions strongly influence bacterial metabolism, where fluctuations in metal availability can shape enzyme function and cellular fitness. In *Streptococcus pneumoniae* (pneumococcus), iron and copper play important yet contrasting biochemical roles. Iron is essential for growth, serving as a cofactor for enzymes involved in DNA synthesis and energy metabolism. In contrast, copper is toxic, as excess intracellular copper promotes mismetallation and induces oxidative stress. While the pneumococcus has dedicated systems for exporting copper, how copper enters cells remains unclear. Here, we show that iron enhances copper accumulation in the pneumococcus, an effect not observed with other trace metals, such as zinc or manganese. Similar results were observed in a capsule-deficient strain. Metal-specific interactions were consistent with copper accessing the cell through iron uptake pathways. In support of this interpretation, the deletion of selected iron transport proteins significantly reduces copper association. Furthermore, RNA-seq analysis revealed upregulation of the copper export system, suggesting that copper triggers the detoxification pathways. Biochemically, we demonstrate that iron substrate-binding proteins can interact with both iron and copper, supporting the idea of mismetallation. Together, these findings highlight how overlapping in metal-binding chemistry can shape metal homeostasis and reveal how essential metal uptake pathways facilitate toxic metal accumulation.

**IMPORTANCE:**

Metal homeostasis is essential for maintaining bacterial metabolism. Disruptions to these pathways can stress cells and reduce viability, thus offering potential avenues for antibacterial therapies. For bacteria at the host-pathogen interface, iron acquisition is well characterized and crucial for survival. In contrast, copper is intrinsically toxic in excess, as reflected by the strong, ubiquitous copper-export systems. Despite this toxicity, the mechanisms by which copper enters bacterial cytoplasmic space, specifically *Streptococcus pneumoniae*, remain poorly defined. Here, we identify a previously unexplored route for copper accumulation, in which copper exploits iron transport pathways to enter the cell. This finding reveals a potential vulnerability in metal homeostasis that may be leveraged to better understand bacterial physiology and the metallo-environment.

## INTRODUCTION

Metals serve as essential structural and catalytic cofactors of enzymes and are required for bacterial growth and proliferation (1–3). Bacteria have complex influx and efflux systems that work to maintain homeostatic levels of metal in the organism. *Streptococcus pneumoniae* TIGR4 (pneumococcus) is a Gram-positive fermentative bacterium adapted to survive and compete in metal-restricted host niches. Of the metals it encounters, iron is essential for growth, structural integrity, and virulence (4–7). Consistent with the requirement for iron is the presence of at least six different iron import systems for the transport of ferric and ferrous iron, and iron captured from host carrier proteins or xenosiderophores, and no known iron export system (8–10). Interestingly, neither pneumococcus nor other Streptococcus species make their own siderophores (11). Structurally, three of these transporters (encoded by *Sp_1032-Sp_1035* [*piaA-D*]*, Sp_1869-1872* [*piuA-D*], and *Sp_1824-1826* [*afuA-C*] genes) are composed of an extracellular membrane-anchored substrate binding protein, two proteins forming a transmembrane channel or permease, and an intracellular ATPase for active transport. In this paper, we focus on these three iron transporters.

In contrast to iron necessity, copper is not required and becomes toxic when it accumulates (12, 13). Copper intoxication results in mismetallation, redox stress, and protein aggregation (14, 15). Copper homeostasis is maintained by the copper export system (*cop* operon), which encodes the copper repressor (*copY*), the chaperone (*cupA*), and the exporter (*copA*) (16–18). Although the mechanism governing copper efflux in pneumococcus is well characterized (19), the routes by which copper enters the cell remain undefined. Currently, no pneumococcal proteins are known to require copper as a structural or catalytic cofactor, making it unlikely that the bacteria have evolved or retained a dedicated copper import system and raising the question of how copper is acquired. While a copper import system has been described in the soil-dwelling environmental Gram-positive *Bacillus subtilis* (YcnJ), this system aids in copper uptake for delivery to the storage protein Csp3, which is important for endospore formation (20). Deletion of the *ycnj* gene led to a 30% decrease in intracellular copper as compared to wild type (21). Homologous systems are absent from streptococci and many other Gram-positive pathogens that inhabit the human host.

The diversity of metal transport systems includes other metals such as manganese, zinc, and calcium, with promiscuity observed among these systems. In streptococcal species, uptake and export of trace metals are primarily facilitated through ATP-binding cassette (ABC) (22). These transporters are coupled to substrate-binding proteins for import or to chaperones for export. For example, the ABC transporter MtsABC in *Streptococcus pyogenes* mediates the uptake of zinc and copper. In this system, both metals compete for a common binding site on the SBP MtsA, while a distinct site has been reported to bind Fe(III). However, deletion of MtsA resulted in significant reductions in zinc and iron levels relative to wild-type cells, suggesting differential transport efficiencies among bound metals (23). Subsequent studies revealed that the same transporter can also bind manganese, further highlighting the metal-binding promiscuity of this system (24).

Similarly, in *S. pneumoniae*, the zinc transporter AdcAII exhibits broad metal-binding capability, interacting with Zn, Co, Mn, Ni, and Cu, as evidenced by metal-dependent shifts in protein melting curves (25). In *Staphylococcus aureus,* overexpression of a manganese transporter leads to copper accumulation, whereas disruption of this transporter via transposon mutagenesis significantly reduced intracellular copper levels (26). In addition, biochemical characterization of the ferrous iron transporter FeoB in *S. aureus* revealed that specific metal-binding regions strongly coordinate copper under physiological pH (27). Collectively, these studies show that different transition metals can associate with metal transport proteins, although the extent to which such interactions result in productive transport varies among transporters and metals. Based on these findings, we hypothesized that transition metal transporters facilitate copper accumulation in *S. pneumoniae*.

Here, we used inductively coupled plasma optical emission spectroscopy (ICP-OES), which detects total metal content, regardless of oxidation status, to quantify that copper accumulation in *S. pneumoniae* is closely linked to iron transport systems. While Zn and Mn do not significantly affect copper accumulation, iron markedly enhances it. This iron-enhanced copper accumulation occurs independently of the negatively charged capsule. Furthermore, by targeting multiple iron import systems, we demonstrate that copper accumulation is fundamentally dependent on iron transport pathways and likely facilitated by promiscuous iron transport systems. Together, these findings provide the first mechanistic insight into how copper accumulates in *S. pneumoniae*.

## RESULTS

**Fig 1:**
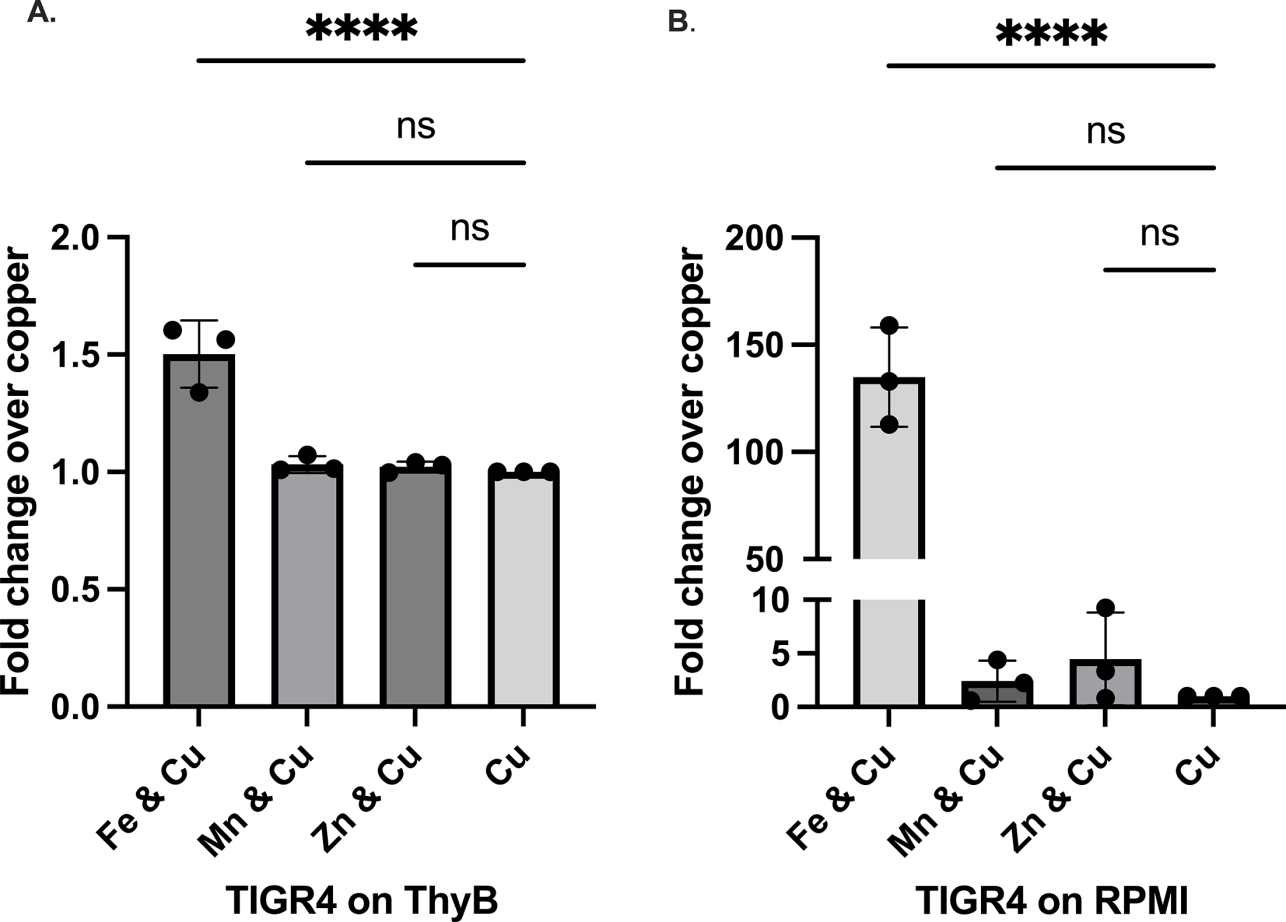
Pneumococcus copper accumulation is greatly enhanced by iron. Impact of metal co-supplementation on copper levels in *S. pneumoniae* in different media. Bar graph showing the fold change in copper levels in combination with iron, manganese, or zinc relative to copper alone. (**A**) Bacterial culture grown on complex media, Todd Hewitt broth with 0.2% yeast extract. A combination of 200 µM copper and 200 µM of the indicated metal with 30-minute exposure. (**B**) Bacterial cultures grown on defined medium RPMI 1640 with modifications as described in Materials and Methods (from here on RPMI_mod_). A combination of 200 µM copper and 200 µM of the indicated metal with 30-minute exposure. Showing three independent biological replicates. Statistical significance was determined by a one-way ANOVA with Dunnett’s multiple comparison test (ns, non-significant; *, *P* < 0.05; **, *P* < 0.01; ***, *P* < 0.001; ****, *P* < 0.0001).

While investigating copper homeostasis in *S. pneumoniae,* we observed that iron exposure enhanced copper accumulation in an iron-dependent manner (Fig. 1), a response not seen with other trace metals relevant to pneumococcus physiology. Additionally, the growth medium used to characterize metal homeostasis strongly influenced the magnitude of this effect. In a complex medium, iron supplementation produced a modest, but significant, 1.5-fold increase in copper accumulation compared to copper exposure alone (Fig. 1A). In contrast, the defined host-adjacent medium RPMI, modified accordingly to Schultz et al. (28) and supplemented with trace metals, produced a 130-fold increase in copper levels (Fig. 1B). To better understand these differences, we quantified trace metals across several media commonly used in our laboratory (Fig. S1). We found statistically significant increases in Mn and Fe concentrations in complex media when compared to defined media.

**Fig 2:**
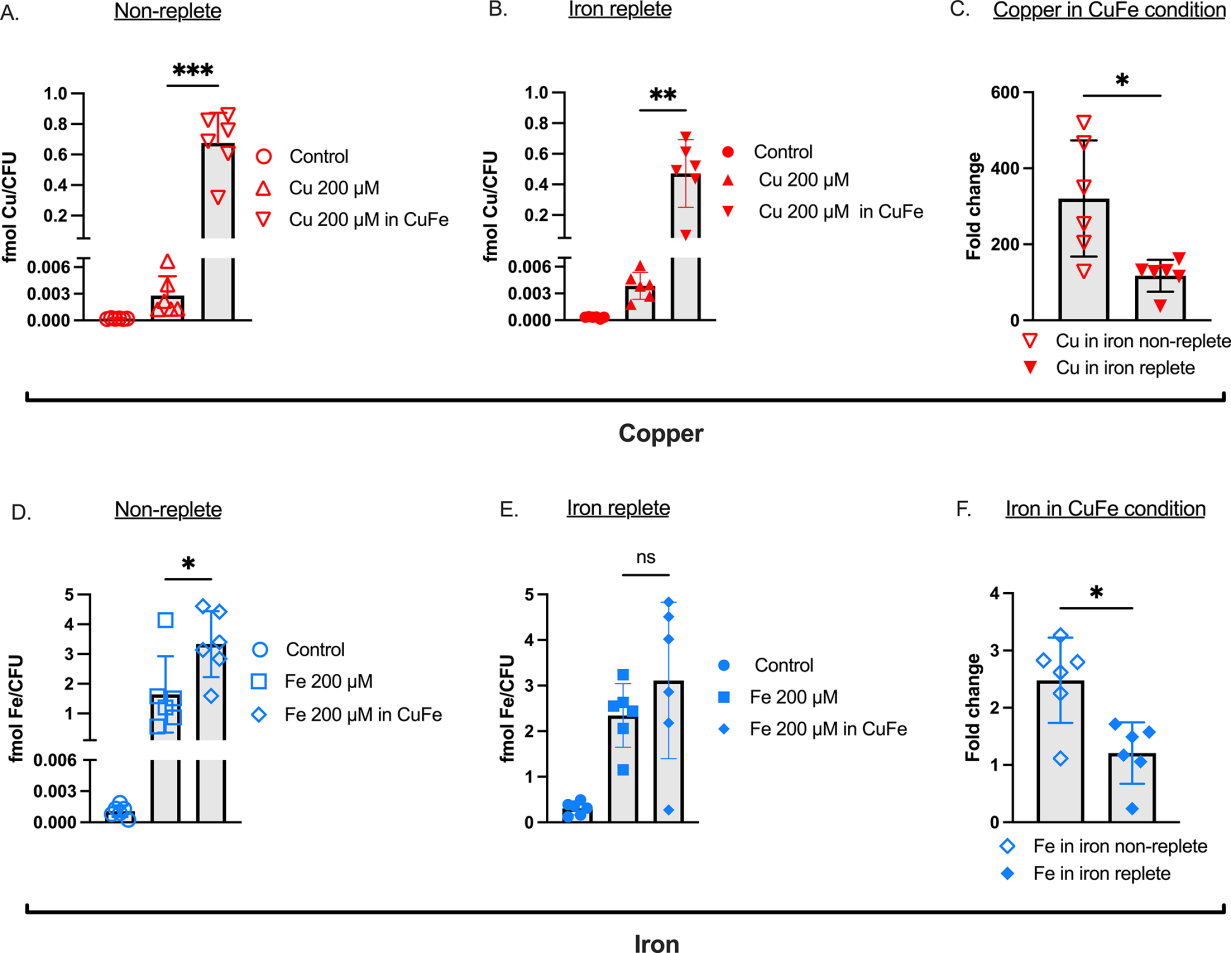
Iron-replete cultures display iron-enhanced copper accumulation. Effect of iron incubation on subsequent copper or iron levels under single and combined metal conditions. Bacteria were grown in non-iron replete (grown on trace metal RPMI medium containing baseline iron) vs iron replete conditions (grown with 100 µM iron). Copper determination during the exponential phase after 30-minute incubation with 200 µM copper and iron in a 1:1 ratio. Bar graph showing iron and copper concentration after exposure to metals with or without prior iron incubation, comparison of fold change between replete and non-replete cultures. (**A**) TIGR4 under non-iron replete conditions displays enhanced copper accumulation when co-exposed with iron. (**B**) TIGR4 under iron-replete conditions displays enhanced copper accumulation when co-exposed with iron. (**C**) Fold change copper comparison between iron-replete and non-replete cultures displays an increase in copper accumulation during non-replete conditions. (**D**) TIGR4 under non-replete conditions displays iron accumulation with or without copper coincubations. (**E**) TIGR4 under iron-replete conditions displays increased levels of iron with or without copper coincubations. (**F**) Fold change in iron accumulation displays an increase in iron levels under non-iron-replete conditions. Statistical significance was determined by a Welch’s t-test (**A, B, D, E**) and a Mann–Whitney test (**C and F**) (ns, non-significant; *, *P* < 0.05; **, *P* < 0.01; ***, *P* < 0.001; ****, *P* < 0.0001).

**Fig 3:**
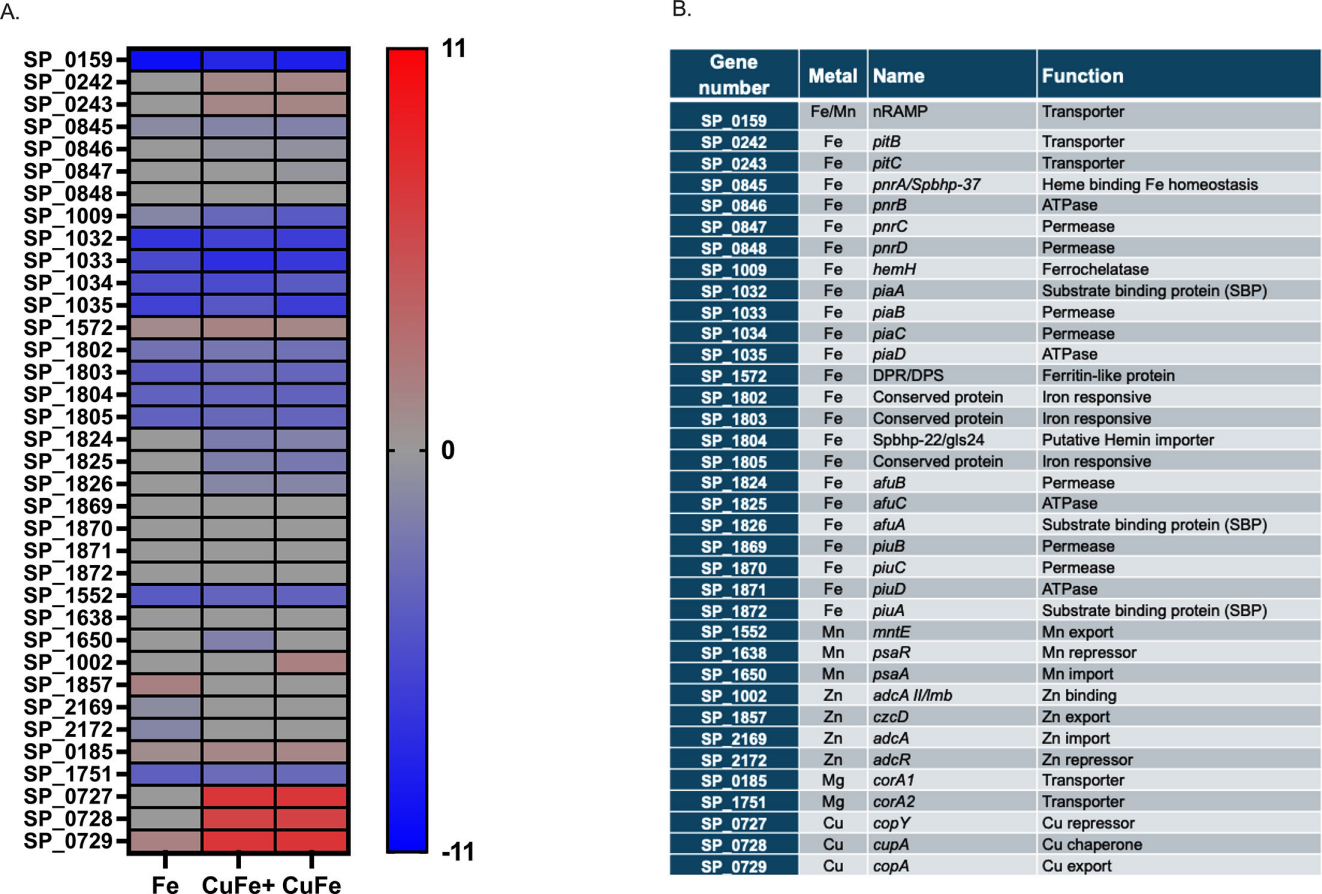
Heat map displaying log_2_ fold change values in gene expression. (**A**) Metal-related transcriptional changes indicating upregulation (red) or downregulation (blue) of genes from bacteria grown to mid-exponential in RPMI_mod_ then exposed to the following conditions: Fe (culture exposed to 200 µM iron), CuFe^+^ (culture grown with 100 µM iron, then exposed to 200 µM copper and 200 µM iron), CuFe (culture exposed to 200 µM copper and 200 µM iron). Gray indicates non-significant changes. (**B**) Table identification of metal-related genes and their purported function.

Given the observation that copper accumulation was enhanced when bacteria were exposed to copper and iron simultaneously, we hypothesized that iron primes the bacteria for copper uptake. To test whether iron needed to be present in parallel or in series for the uptake of copper, cultures were grown with 100 µM iron (replete) or not (iron native RPMI_mod_), followed by exposure to iron, copper, or both (Fig. S2). Cultures pre-exposed to iron during the growth period did not exhibit enhanced copper accumulation as compared to non-replete iron cultures (Fig. 2A through C); yet, independent of preexposure, both non-replete (RPMI_mod_) and replete iron cultures continued to acquire both metals when offered simultaneously (Fig. 2A, B, D, and E). However, cultures pre-exposed to iron showed a significant decrease in both the copper fold change and the iron fold change compared with non-iron-replete cultures (Fig. 2C and F), suggesting downregulation of their iron acquisition pathways, which was confirmed with RNAseq (Table S1 and Fig. S3 and Fig. 3A and B). This result indicates that iron alone does not induce a lasting cellular state that promotes copper accumulation. Instead, these findings support a model in which the simultaneous exposure to both metals is required to enhance copper accumulation, consistent with an acute, co-exposure-dependent process rather than sustained iron-promoting copper accumulation.

### Copper accumulation is not capsule-associated and engages the copper export system

**Fig 4:**
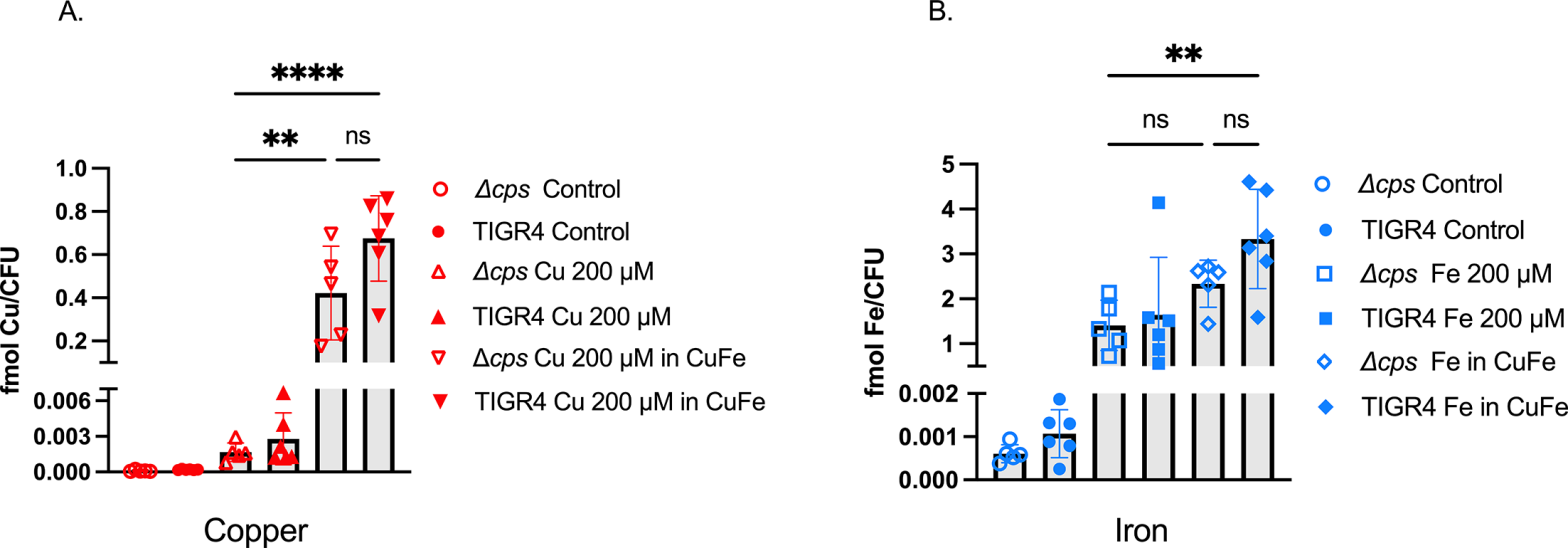
TIGR4 capsule null display iron-enhanced copper accumulation. Effect of capsule deletion on copper and iron levels after copper or iron exposure. Bar graph showing capsule-null strain or TIGR4 exposed to 200 µM copper, iron, or a combination for 30 minutes. Metal content comparison against an untreated control. (**A**) Showing fmol copper in capsule-null strain and TIGR4 after 30 minutes of exposure to the indicated conditions. (**B**) Showing fmol iron in capsule-null strain and TIGR4 after 30 minutes of exposure to the indicated conditions. Statistical significance was determined with a one-way ANOVA with Tukey’s multiple comparison (ns, non-significant; *, *P* < 0.05; **, *P* < 0.01; ***, *P* < 0.001; ****, *P* < 0.0001).

A prominent feature of pneumococcus is its polysaccharide capsule, which carries a negative charge and zeta potential (29, 30). This characteristic raised the possibility that the observed phenotype could be explained by the positively charged copper ions adhering to the negatively charged capsule instead of entering the cell. To address this concern, we repeated the experiments using an isogenic capsule-null strain, ∆*cps* TIGR4. When pneumococcus is exposed to copper, we detected increased copper levels in both the wild type and the capsule-null mutant. Furthermore, when both strains were exposed to copper and iron at a 1:1 molar ratio, iron greatly enhanced copper accumulation, resulting in a significant increase compared to copper alone (Fig. 4A). Additionally, there is no statistical difference in copper accumulation between the capsule null and the wild-type strain. These findings indicate that copper association with the wild-type strain is not capsule-dependent but reflects copper internalization. Iron levels were also consistent between the capsule mutant and wild type and only reached statistical difference when in combination with copper (Fig. 4B).

Another barrier to copper internalization is the bacterial membrane. To further support intracellular copper sensing, we performed RNAseq under the same exposure conditions. After 30 minutes of exposure to copper and iron at a 1:1 molar ratio, the most upregulated set of genes was the copper export system, which increased by 7 log2-fold over control (Fig. S3), followed by thioredoxin genes at 3.6 log2-fold change. The copper export system is repressed by the CopY regulator and requires direct copper interaction for operon activation (17, 31, 32). The copper concentration in RPMI_mod_ was found to be 130 nM, which is low but sufficient to maintain baseline expression of the copper export system, thereby avoiding an extreme On/Off regulatory state. Additionally, the upregulation of thioredoxins suggests a cellular response to oxidative stress induced by copper exposure. Together, these data strongly support that copper is sensed intracellularly in pneumococcus. In contrast to the dynamic changes that we observed in the transcriptome, the metal proteome remained mostly unchanged in the 30-minute time frame, which provides insight into the continued increase in metal uptake (Table S1 and Fig. S4).

### Iron transporters contribute to cellular copper accumulation

**Fig 5:**
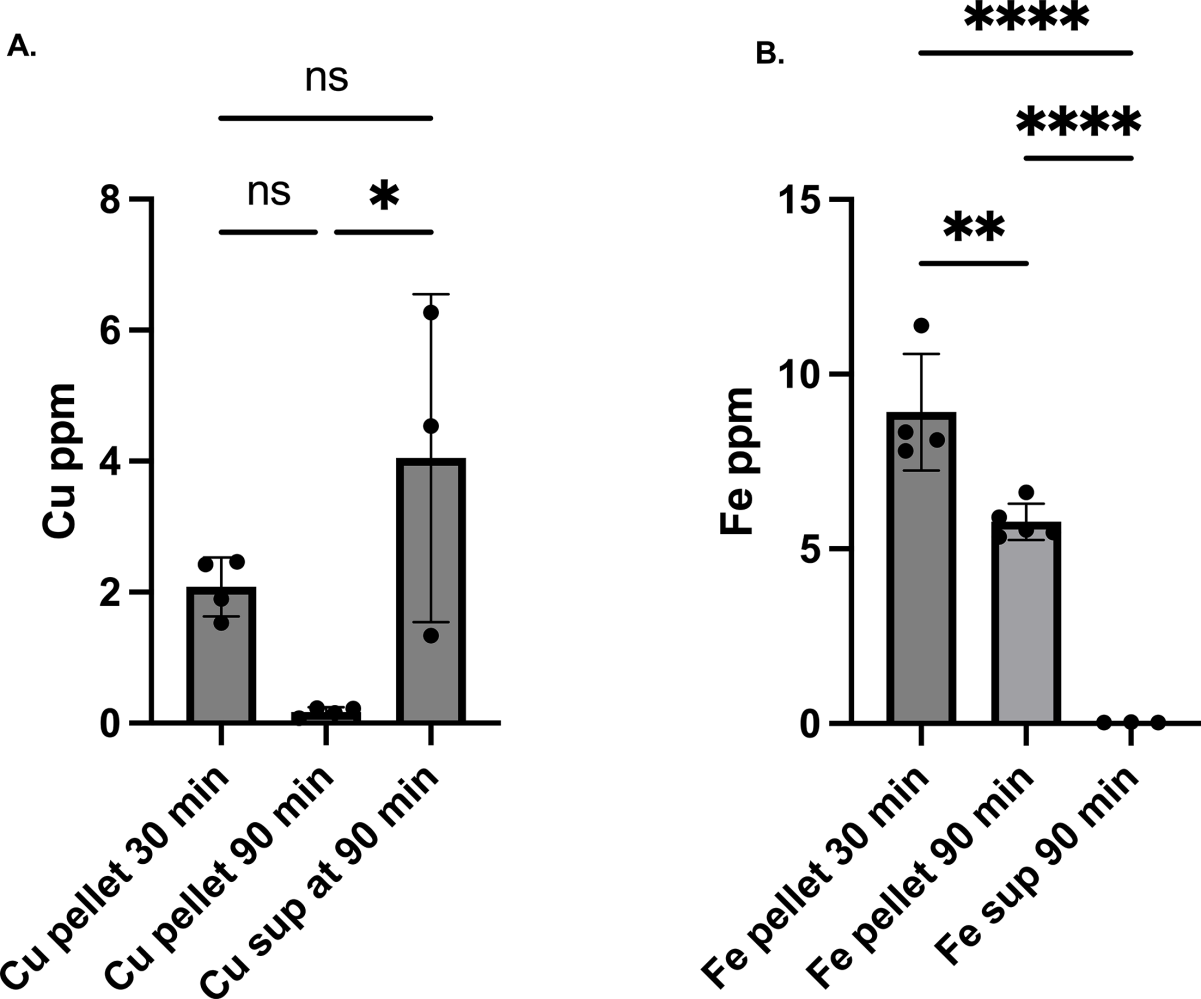
Metal clearance after transient iron and copper exposure. Bacteria were grown on RPMI_mod_ and exposed to a combination of 200 µM copper and iron for 30 minutes, followed by centrifugation and resuspension in fresh medium. Liquid samples were taken at intervals to determine CFU and metal content. (**A**) Copper levels at 30 minutes post-exposure or 90 minutes after metal removal in pellet and filtered supernatant (sup). (**B**) Iron levels at 30 minutes post-exposure or 90 minutes after metal removal in pellet and filtered supernatant (sup). Statistical significance was determined by a one-way ANOVA with Tukey’s multiple comparison (ns, non-significant; *, *P* < 0.05; **, *P* < 0.01; ***, *P* < 0.001; ****, *P* < 0.0001).

To determine whether iron-enhanced copper accumulation could alternatively be explained by impaired copper export rather than iron-enhanced accumulation, we monitored copper and iron clearance following the removal of extracellular metals. Cultures were exposed to excess copper and iron for 30 minutes, after which cells were transferred to fresh medium and CFU counts and intracellular metal content were monitored for 90 minutes. CFU counts showed non-significant changes during this time (Fig. S5). Iron decreased from 8 ppm to 5.5 ppm, resulting in a 23% decrease over the 90 minutes, while copper decreased from 2 ppm to below 0.5 ppm, corresponding to an 85% reduction in copper content over 90 minutes (Fig. 5A and B). Concomitantly, we detected an increase in copper signal in the supernatant at 90 minutes, combined with a decrease in the detection of iron in the supernatant at 90 minutes (Fig. 5A and B). We attempted to measure the metal content in the supernatant at 30 minutes, but the continued presence of the original metal exposure in the medium confounded the results. While CFU counts remained stable following metal removal, we cannot exclude the possibility of cellular turnover during this period. However, such turnover could not readily explain the strongly asymmetric clearance kinetics observed for copper versus iron. These data are consistent with efficient copper export following the removal of extracellular metals and provide functional support for the transcriptomics signatures of copper export upregulation and iron transport downregulation (Fig. 3A).

**Fig 6:**
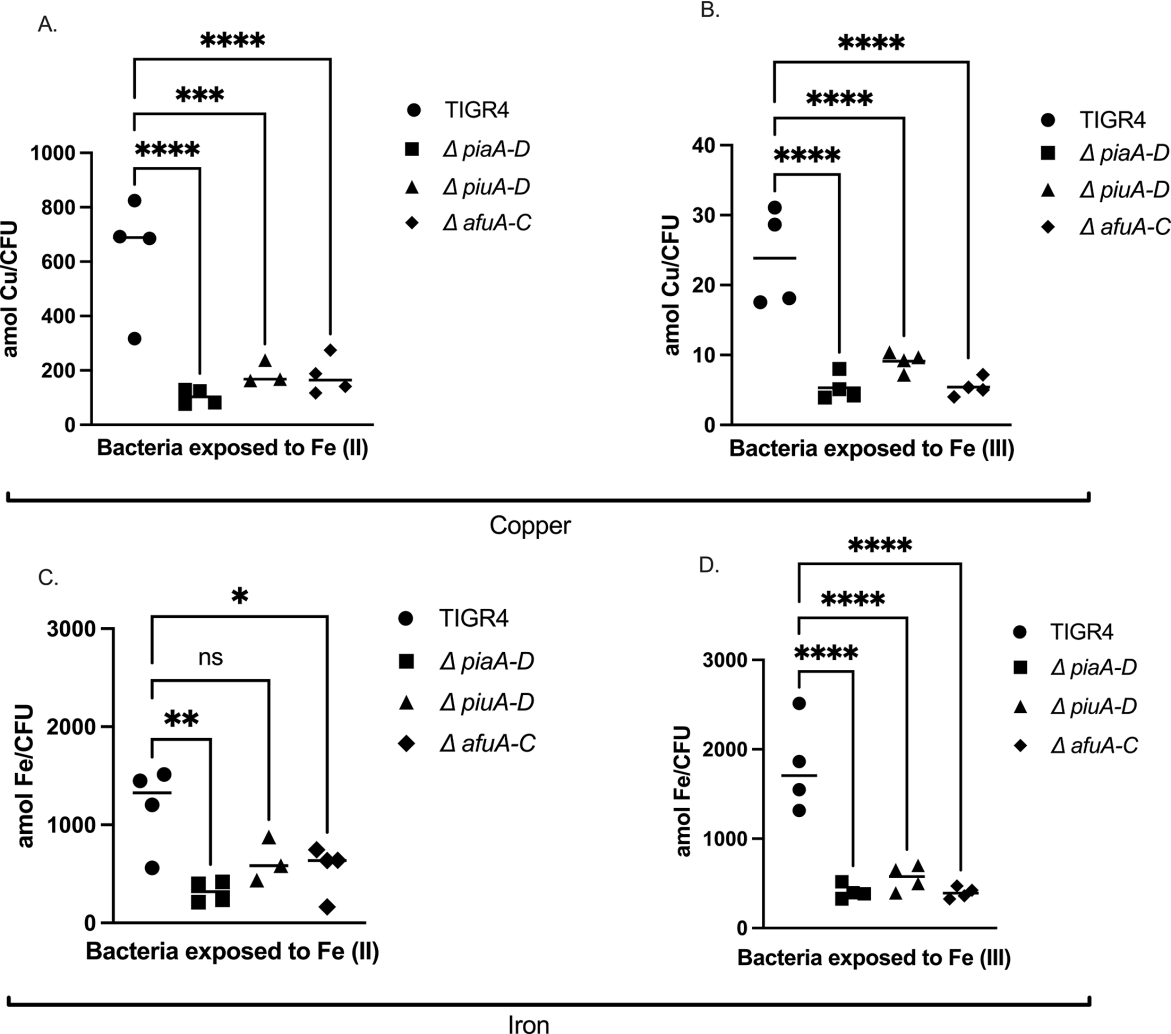
Loss of iron transporters reduces iron and copper accumulation. Dot plot showing metal content in wild type and iron-transport mutants. Bacteria were grown in RPMI_mod_ then exposed to 200 µM copper, iron, or copper and iron for 30 minutes. (**A and B**) Levels of copper in TIGR4 or iron transport mutants during iron–copper coincubations. (**A**) Coincubations with Fe(II) and Cu(II). (**B**) Coincubations with Fe(III) and Cu(II). (**C and D**) Levels of iron in TIGR4 or iron transport mutants during iron-copper coincubations. (**C**) Coincubations in Fe(II). (**D**) Coincubations in Fe(III). Statistical significance was determined by a one-way ANOVA with Dunnett’s multiple comparison (**A, C, and D**) and one-way ANOVA with Tukey’s multiple comparison (**B**) (ns, non-significant; *, *P* < 0.05; **, *P* < 0.01; ***, *P* < 0.001; ****, *P* < 0.0001).

Having established that iron enhances copper association specifically during co-exposure and that copper accumulation is not attributable to impaired export, we next sought to directly test the contribution of iron transport systems to copper association. To this end, we generated iron transport system knockouts (∆*pia,* ∆*piu,* and ∆*afuA-C*) and quantified both iron and copper acquisition. Iron transport mutants exhibited a growth delay when grown in supplemented RPMI (Fig. S6), prompting us to collect samples at the same optical density during the exponential phase, minimizing the impact of the lag phase and preventing changes brought by different metabolic states. Iron transport mutants were still able to acquire both iron and copper when supplied individually or in combination, and this phenotype persisted, regardless of the oxidation state of the iron provided (Fig. 6 and Fig. S7). Iron mutants displayed a significant reduction in copper content when compared to wild-type pneumococcus on the same genetic background (Fig. 6A through B). Likewise, iron content was decreased in iron transport mutants (Fig. 6C through D).

The combination of copper and iron can promote compounding oxidative effects over time, which can lead to membrane damage and ion leakage. An alternate hypothesis for our observation is that membrane damage promotes copper accumulation by disrupting the membrane. To explore this hypothesis, we analyzed ICP-OES data to examine changes in other metal ions. We postulated that if membrane damage were occurring, it would cause overall changes in intracellular metals across all exposed bacteria, regardless of mutation status. Wild type and capsule mutant display a statistically significant accumulation of Zn, Mn, Mg, Ca, and Na after exposure to iron and copper combination (Fig. S8A through E). In contrast, the iron transport mutants have a different response after simultaneous exposure to iron and copper. Mn, Mg, and Ca failed to reach statistical significance but show a trend toward higher levels only in the ∆*piu* system mutant (Fig. S8B through D), while the ∆*pia* system mutant displayed a trend toward higher Mn levels. These differences in bacterial responses suggest a targeted transcriptional adaptation to the decrease in iron uptake pathways caused by the deletion of iron transporters. Furthermore, transcriptionally, we can confirm that the increase in Mn and Mg observed in the wild type is due to downregulation of the Mn and Mg exporters, suggesting efforts to maintain metal homeostasis (Fig. 3A). This difference in metal accumulation between all the mutants and wild type suggests that the changes we observed in metal concentrations, including iron and copper, are associated with transport rather than catastrophic membrane permeability loss.

**Fig 7:**
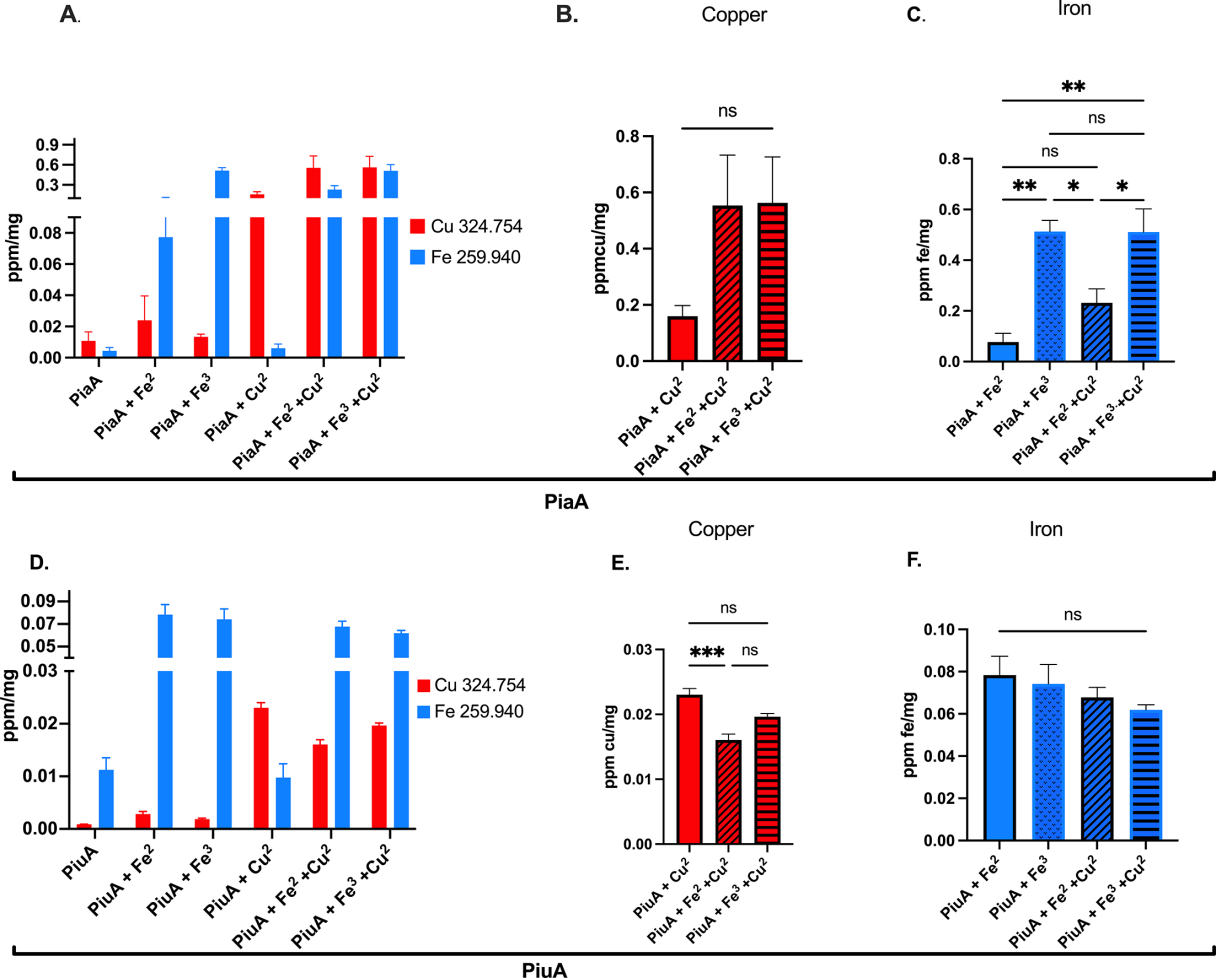
Substrate-binding proteins from iron transporters bind copper and iron *in vitro*. Bar graphs showing metal content via ICP-OES after exposure to Cu, Fe, or a combination of both metals, followed by size exclusion chromatography. (**A**) Showing the substrate-binding protein made from Sp_1032 (PiaA) under different conditions. (**B**) Comparing the copper levels with or without iron co-exposures. (**C**) Comparing the levels of iron at two distinct oxidation states with or without copper co-exposure. (**D**) Showing the substrate-binding protein made from Sp_1872 (PiuA) under different conditions. (**E**) Comparing the copper levels with or without iron co-exposure. (**F**) Comparing the levels of iron at two distinct oxidation states with or without copper co-exposure. Statistical significance was determined via one-way ANOVA with Tukey’s multiple comparison (ns, non-significant; *, *P* < 0.05; **, *P* < 0.01; ***, *P* < 0.001; ****, *P* < 0.0001).

To assess whether iron substrate-binding proteins interact with copper, we purified the iron substrate-binding proteins PiuA and PiaA using affinity chromatography and confirmed purity by SDS-PAGE (Fig. S9). Purified proteins were incubated with copper, iron, or a combination of both metals, followed by size-exclusion chromatography to remove metal-protein aggregates. Protein-metal complexes were subsequently digested by acid and heat treatment and analyzed by ICP-OES. After purification by FPLC, we found that PiaA had trace levels of both iron and copper under non-metal-exposed control conditions. Incubating the purified protein with either Fe(II) or Fe(III) increased the levels of iron associated with the protein (Fig. 7A). Although the copper-only control showed iron as well, we detected both iron and copper associated with PiaA when both metals were present, with levels 10-fold higher than baseline levels of metal association. Iron levels did not seem to significantly influence the levels of copper present when incubated together (Fig. 7B). While the protein appeared to bind Fe(III) significantly better than Fe(II), that trend remained even when incubated with copper (Fig. 7C). Similarly, PiuA contained trace levels of iron and copper after purification via FPLC. This protein also bound Fe(II) and Fe(III) at comparable levels (Fig. 7D and F), suggesting some flexibility in its metal binding. Copper levels were significantly reduced when incubated with Fe(II), while coincubation with Fe(III) did not reach significance. The protein also bound both copper and iron when presented together. Taken together, the two iron systems substrate-binding proteins can bind both iron and copper either sequentially or simultaneously.

We performed a multiple sequence alignment using Clustal (33) of the characterized ferrichrome transporter FhuB against the permease subunits PiaB and PiaC to compare conservation of binding- and gating-associated regions. This analysis was used to assess whether residues implicated in substrate coordination and translocation show conservation of properties consistent with metal-coordinating capacity and potentially for weak, non-canonical transition metal interactions. The alignment revealed that the permease retains the hydrophobic transmembrane architecture characteristic of a ferrichrome-associated transporter, suggesting conservation of overall transport fold and gating mechanism. Substitution of a conserved HDQ region with an RYE located at the 177–182 motif potentially alters local coordination chemistry while maintaining a polar substrate interaction environment (Fig. S10).

### Functional implications

Iron is critical for pneumococcus growth and survival in the human host. Consequently, losing any number of iron transporters confers a loss of fitness to the bacterium. This is evident from the decrease in adherence and invasion of A549 cells after deletion of *spd_1590* (reported hemin transporter), while mice inoculated with *spd_1609* knockout (*afuA*, Fe(III) transporter) showed better survival (7, 34). Likewise, iron mutants have a longer lag phase during *in vitro* growth (Fig. S6). These experiments highlight the significance of maintaining multiple iron acquisition systems in the pneumococcus and how disruption of these undermines the pathogen’s ability to compete and persist. Given these qualities, we then interrogated whether losing any iron acquisition pathway could confer some type of benefit?

**Fig 8:**
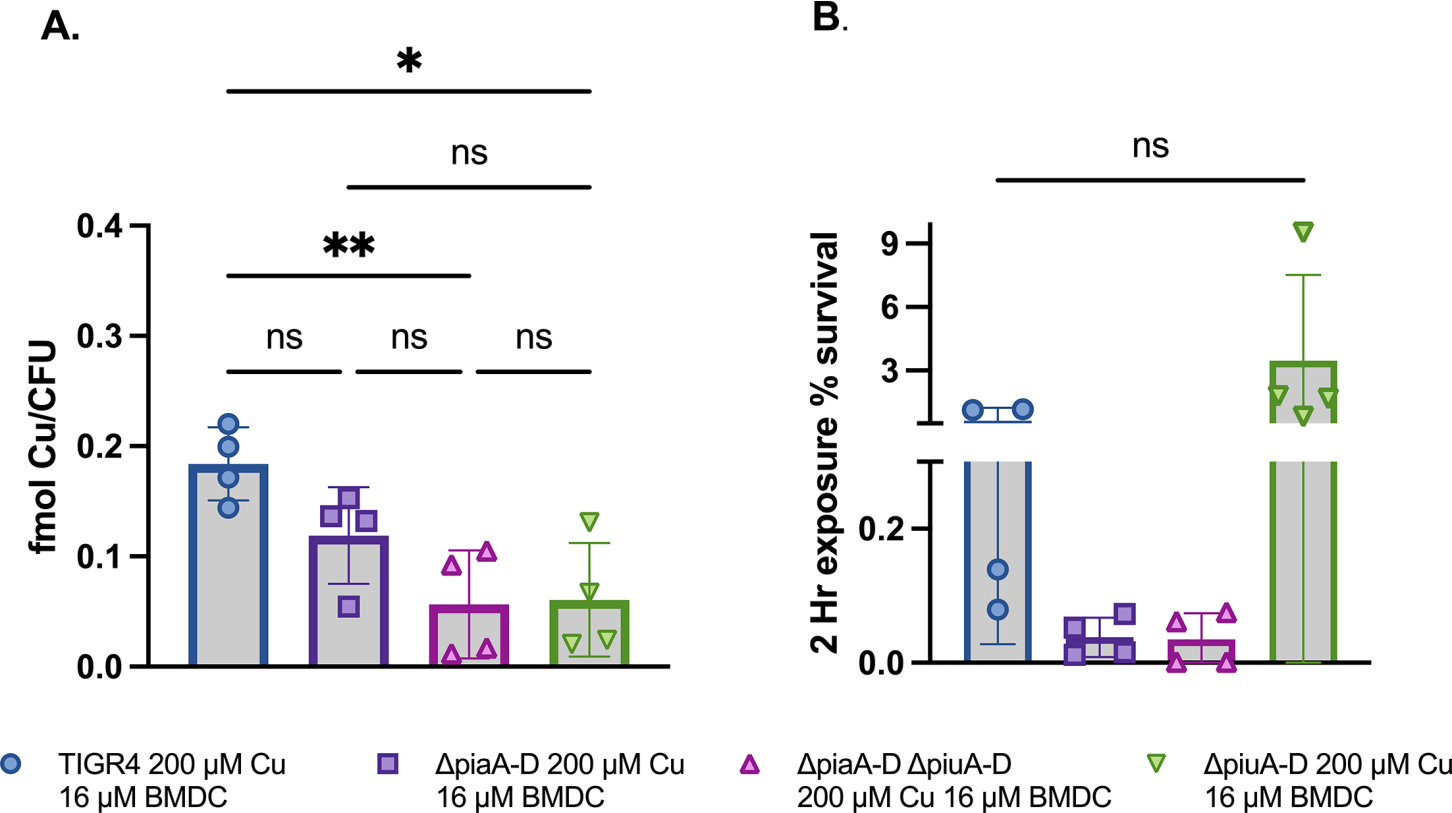
Iron mutant accumulates less copper, facilitated by BMDC. TIGR4, *∆piaA-D, ∆piuA-D,* or double iron transport mutant (*∆piaA-D/piuA-D*) cultures were grown on ThyB and exposed to 200 µM copper and 16 µM BMDC for 30 minutes or 2 hours. (**A**) Copper content was measured via ICP-OES 30 minutes post-exposure to the metal-compound condition. (**B**) Percent survival based on the initial inoculum was determined at 2 hours of continued metal-compound exposure. Statistical significance was determined via one-way ANOVA with Tukey’s multiple comparisons (ns, non-significant; *, *P* < 0.05; **, *P* < 0.01; ***, *P* < 0.001; ****, *P* < 0.0001).

Our lab has published on copper-dependent antimicrobials (35–38). These compounds work through different mechanisms depending on the bacteria being targeted. Previous work on DMDC (the parental compound of BMDC) demonstrates that at least in pneumococcus, DMDC accelerates the rate at which copper becomes internalized, resulting in a loss of viability (36, 38). One hypothesis is that dithiocarbamate compounds work by mimicking siderophores, but when supplied with copper, they increase copper instead of iron, and, given this work, we wondered whether the loss of iron transport would reflect a decrease in copper-dependent killing. We exposed the iron transport mutants to the copper-dependent antimicrobial for 30 minutes, then measured intracellular copper content in comparison to the wild type. With these conditions, ∆PiuA-D showed a major contribution in decreasing intracellular copper concentration when this antimicrobial was used (Fig. 8A). To determine whether this decrease in copper accumulation would translate into increased survival over time, we increased exposure to 2 hours and quantified bacterial survival. There was no statistical difference in survival after continuous exposure to the combination of copper with BMDC (Fig. 8B), suggesting the rate of copper delivery mediated via BMDC must reach an equilibrium that overcomes the ability of the copper exporter to remove toxic metal from the interior.

**Fig 9:**
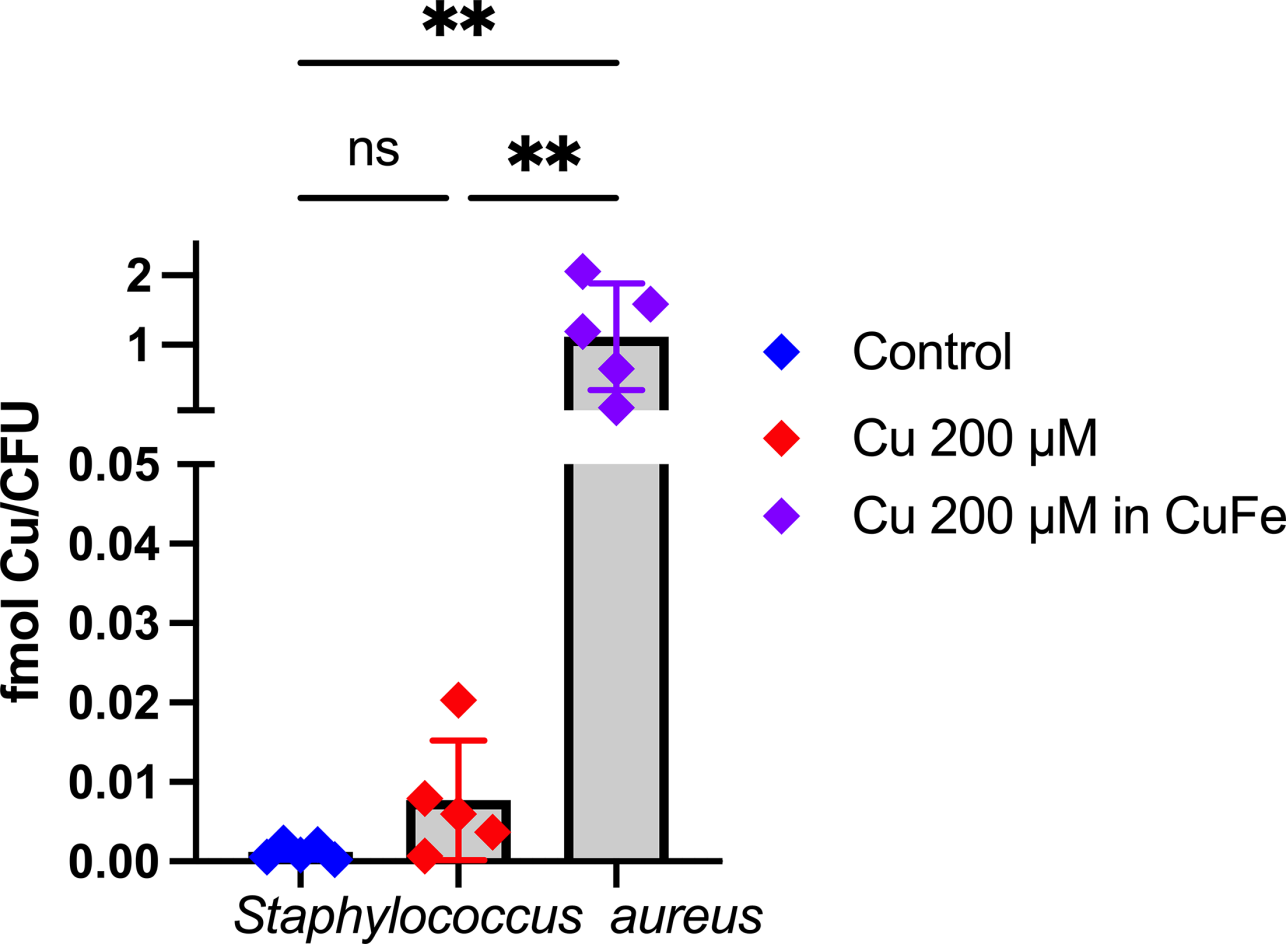
Iron promotes copper uptake in *Staphylococcus aureus*. Bar graph showing copper content as determined by ICP-OES after 30 minutes incubation with 200 µM of copper, iron, copper and iron, or control. Statistical significance was determined via one-way ANOVA with Tukey’s multiple comparisons test (ns, non-significant; *, *P* < 0.05; **, *P* < 0.01; ***, *P* < 0.001; ****, *P* < 0.0001).

*Staphylococcus aureus* is another Gram-positive bacterium that colonizes the nares of roughly 30% of the healthy population (39). It is also susceptible to copper stress when provided in excess and just like pneumococcus has no requirement for copper. In fact, copper causes stress responses that alter carbon metabolism through direct interaction with glyceraldehyde-3-phosphate dehydrogenase (GAPDH) and the catabolite control protein (CcpA) (40, 41). Recently, it was discovered that under zinc limitation, the metallophore staphylopine promiscuously binds and imports copper into staphylococcal cells, causing copper intoxication (42). Although this bacterium produces the siderophores staphyloferrin A and B for iron acquisition from the host (43), we pondered whether copper entry could also occur in this bacterium when exposed to copper and iron. As such, we found a significant increase in copper associated with the bacteria in the presence of iron compared to the copper-only condition (Fig. 9).

## DISCUSSION

As a survival strategy, bacteria use a network of internal and external sensors that relay information about the environment, dictating single and collective behaviors that enable bacteria to grow and thrive in their environment. Consequently, the composition of the growing medium plays a critical role when interrogating biological responses to stimuli (44). This effect is clearly illustrated in Fig. 1 and Fig. S1. Complex microbiological media such as ThyB, which are composed of animal organ infusions and enzymatic digest of proteins, inherently contain variable and undefined concentrations of trace metals as well as other small molecules such as heme or hemin. In contrast, defined media such as RPMI 1640 consist of measured components, including controlled concentrations of inorganic salts and trace metals, resulting in more consistent nutrient availability. Figure S1 shows the variability in trace metal content between ThyB and RPMI_mod_. Because trace metals are crucial for bacterial growth and development, and maintaining metal homeostasis is essential for bacterial fitness, differences in media composition can significantly influence bacterial responses. To minimize confounding effects, we characterized bacterial responses to copper exposure in a host-adjacent medium (media that host cells traditionally can grow in), RPMI 1640, modified as described in reference 28 and supplemented with trace metals (see Materials and Methods). Although the same concentrations of base metals were added to the growth medium, random testing revealed variability in metal concentrations between media preparations, as shown in Fig. S1. ICP-OES analysis of different commonly used bacterial growth media showed disparity in important trace metals, such as zinc and iron. Such variability is particularly significant given the central role of iron in bacterial growth, morphology, metabolism, and virulence (4, 11, 45–47). Iron acquisition and regulation represent a major energetic investment for bacteria, reflecting the selective pressure imposed by iron limitation in host environments. As such, experimentation in different media can yield novel insights into bacterial physiology (44).

*S. pneumoniae* possesses a well-known copper exporter; however, the pathway by which copper is imported into the bacterium and many other Gram-positive microbes at the host-pathogen interface has not been identified (48). While investigating metal homeostasis in the context of copper exposure, we grew bacteria in the presence of excess copper and another metal at a 1:1 ratio. Metal acquisition in bacteria requires careful regulation to maintain homeostasis and prevent the harmful consequences of mismetallation or oxidative stress. Testing metal homeostasis can be performed by increasing metal levels to create a concentration gradient or by deleting metal exporters to force intracellular accumulation. Because copper accumulation is toxic, we avoided disrupting copper export and instead stimulated copper import by briefly elevating the copper concentration in the growth medium. Under these conditions, we found that concurrent exposure to copper and iron enhanced copper accumulation. This effect was more pronounced in the host-adjacent medium RPMI, suggesting that the interaction between these metals may be important under infection conditions where both metals might be present, even if not always readily accessible.

Metal biosorption by extracellular polysaccharide substances or capsule-producing bacteria is a strategy in the bioremediation industry, as these polysaccharides bind and immobilize metals (49–52). After learning that copper levels continued to increase during iron and copper exposure, we questioned whether the detected copper was associated with the pneumococcal capsule instead of in the intracellular space. We conducted similar experiments using a capsule null mutant in the TIGR4 genetic background and confirmed that, like TIGR4, exposure to a combination of copper and iron also increases copper in the capsule null (Fig. 4A). Moreover, comparing metal concentrations between the capsule-null and wild-type strains shows there is no significant difference between the capsule-null and isogenic strain, indicating that the pneumococcal capsule is not the major contributor to copper signal. Furthermore, transcriptome analysis under copper and iron exposure confirmed an upregulation of copper export genes under copper exposure, followed by upregulation of thioredoxins, confirming that copper reached the intracellular space in the pneumococcus and not stuck in the membrane or capsule.

The iron-replete experiment strengthened the connection between iron availability and enhanced copper accumulation (Fig. 2). Both culture types acquired copper and iron when offered together, but the magnitude of response differed substantially. Iron non-replete cultures showed a greater relative increase in copper accumulation, reflected in a significantly higher fold change (Fig. 2C). Whereas iron-replete cultures display only a modest increase, consistent with a downregulation of iron acquisition pathways. Transcriptomic data confirmed this, revealing reduced expression of iron-transport genes in iron-replete cells exposed to both copper and iron. This result indicates that, while absolute copper levels rise in both conditions, the relative responsiveness of iron-non-replete cells is greater. Biologically, these findings suggest that prior iron exposure does not induce a lasting state that promotes copper accumulation. Instead, the requirement for simultaneous copper and iron exposure suggests a model in which the presence of both metals enhances copper accumulation.

Iron substrate-binding proteins have been canonically characterized for iron acquisition alongside Fe(III) chelates, such as hydroxamate or catecholate siderophores (8, 53). Therefore, we sought to address this experimental variable. Iron speciation in liquid is sensitive to redox conditions and oxygen availability (54). Under fully aerated conditions, Fe(II) is rapidly oxidized to Fe(III); however, in capped culture tubes lacking active aeration, oxygen becomes progressively limited as bacterial growth proceeds. Although pneumococci do not use oxygen as a terminal electron acceptor, they can utilize oxygen during metabolism to support fermentation via the activities of LctO and SpxB (55–57). Under these conditions, we hypothesize that the interconversion of Fe(II) to Fe(III) does not reach equilibrium, resulting in the coexistence of multiple iron species in the growth medium formed through interaction with medium components such as amino acids or buffering components. Consequently, iron is present as a dynamic and chemically heterogeneous pool that includes soluble Fe(II) as well as weakly or transiently formed Fe(III) complexes without the need for canonical siderophores. This heterogeneous iron pool provides a solution to bacterial iron requirements. The same logic can be applied to added Fe(III), and accordingly, we observed similar levels of iron accumulation in the wild type following Fe(II) or Fe(III) incubations, suggesting the bacteria are not iron-starved (Fig. 6C through D). Consistent with this, others have reported bacterial survival under Fe(III) conditions (6).

Having established that iron enhances copper accumulation during metal co-exposure, we next determined whether iron transport systems contribute directly to copper accumulation by generating deletions of iron import systems. Iron acquisition in pneumococcus is highly regulated; therefore, we deleted individual importers. We observed that iron transport mutants retained the ability to accumulate copper in an iron-dependent manner, like wild-type cells. However, relative to wild-type cells, these mutants exhibited a significantly reduced copper accumulation (Fig. 6A and B). Together, these findings indicate that intact iron transport systems are required for maximal copper accumulation, supporting a model in which iron import systems contribute to copper buildup. Notably, the copper-dependent antimicrobial test also demonstrated a significant decrease in the concentration of copper, specifically after *piuA-D* deletion. The loss of iron transporters might appear advantageous by limiting copper-dependent toxicity; however, both our data and other studies demonstrate that disruption of iron uptake systems is detrimental to bacterial fitness, indicating that any reduction in copper susceptibility comes at a substantial physiological cost.

Furthermore, to determine whether iron substrate-binding proteins are involved in copper binding, we affinity-purified the substrate-binding proteins PiuA and PiaA and then ran them over a desalting column for buffer exchange. Both proteins bound iron when either the Fe(II) or Fe(III) oxidation states were added, albeit with different preferences (Fig. 7C and F). The proteins also bound copper, and most importantly, PiuA and PiaA bound copper and iron together. We acknowledge that protein-metal-binding assays alone do not constitute conclusive evidence of metal coordination within a binding pocket. Nevertheless, the data are consistent with an interaction between protein and metals. Specifically, we observed statistically significant oxidation-state-dependent differences in metal association, with a clear preference in PiaA for Fe(III) (Fig. 7A and C). Notably, this preference is maintained upon the addition of copper, indicating that Fe(III) binding is not displaced under competitive conditions. In contrast, copper association with PiaA remains unchanged in the presence of iron, suggesting copper was not perturbed during iron binding under our experimental conditions. In contrast, PiuA bound iron equally, and copper did not disrupt iron binding, but copper decreased in the presence of iron (Fig. 7E), suggesting slight binding site competition or destabilization of the copper-binding site. While these assays do not demonstrate active transport, they provide a biochemical basis for the iron-dependent copper accumulation observed *in vivo* and provide important confirmation that interactions between iron substrate-binding proteins and the metals copper and iron are possible, at least *in vitro*. Demonstrating that the iron substrate-binding protein can accommodate copper either alone or in combination with iron, which supports a model in which iron transport machinery may directly contribute to copper accumulation.

Together, our findings suggest a model in which iron substrate-binding proteins are adventitiously mismetallated by copper. The Irving-Williams series order of stability of metal complexes describes the increasing stability of complexes formed by divalent first-row transition metals. Showing increasing stability from left to right in the periodic table, with copper complexes becoming the most stable (58–60). This trend is driven by electronic structure, as copper’s d9 configuration allows stabilization when interacting with other ligands. This biophysical manifestation dictates that proteins are more likely to bind copper than any other metal. In a biological context, this creates a fundamental challenge, as proteins must selectively bind the correct metal despite copper’s intrinsic competitive advantage. This is one reason why cellular systems tightly regulate and sequester metals.

Cells rely not only on the tight regulation of metal but also on the chemical selectivity of protein metal-binding sites to achieve correct metallation. The strength and specificity of protein-metal interactions depend on the identity and arrangement of coordinating amino acid residues. For iron or copper, residues containing nitrogen, sulfur, and oxygen donors predominate in coordination chemistry with different affinities for the metal. Strong copper ligands include cysteine and histidine, as the thiolate groups form stable interactions with copper, while the imidazole ring of histidine can stabilize both copper oxidation states. Methionine, though less reactive than cysteine, also contributes sulfur coordination, becoming important during transport (61, 62). In contrast, iron tends to favor oxygen donor ligands such as those carboxylate groups found in aspartate and glutamate. Histidine can also bridge and stabilize some of those motifs found in heme and non-heme proteins. Tyrosine can also participate in iron chemistry due to its phenolic oxygen (63, 64). Weaker interactions are mediated via residues like serine and threonine or backbone carbonyls. Water molecules and different solvents complement these weaker interactions. The SBP data show that binding of copper to the proteins does not interfere with iron binding (Fig. 7B, C, E, and F), suggesting iron coordination supersedes copper (as expected), likely through a weaker copper residue interaction.

Dedicated copper transporters possess CXC or CXXC motifs that confer high affinity and selective copper binding. These cysteine-rich motifs are characteristic of proteins specialized for copper trafficking, such as Ycnl in *B. subtilis*. In contrast, the SBP PiaA lacks these conserved motifs, indicating its main function is not copper transport. Nevertheless, the protein contains metal-binding domains capable of coordinating transition metals, raising the possibility that copper weakly interacts with or near the binding site despite the absence of canonical copper-binding motifs (Fig. 7A and D).

Given that several of the residues involved in iron coordination possess side chains capable of transient metal interactions, it is plausible that these same features could permit copper association under certain conditions. Our data suggest that in the presence of iron chelates, copper does not directly compete for the primary binding site but instead interacts peripherally with an adjacent metal-coordinating residue. One possibility is a cluster of residues between 136 and 141 containing hydrophilic EDY residues being stabilized by a D at position 111 in PiaA, as these are in a flexible region of the protein. Another potential group of residues is located between positions 86 and 96 and contains D and E with weak copper-binding properties. Such a configuration could allow copper to transiently associate with the iron-loaded complex, effectively “hitchhiking” during the conformational transition that leads to substrate transfer. This weak, secondary coordination would be consistent with the absence of a high-affinity copper-binding motif, while still enabling low-efficient copper uptake when iron is present.

Cheng et al. identified the residues in ferrichrome interacting with the substrate-binding protein PiaA (53). The amino acids Tyr225 and Arg231 pull the iron moiety via hydrogen bonding from the ferrichrome molecule, while seven residues from the N and C termini are involved in stabilizing and ensuring a high affinity between ferrichrome and PiaA (53). Work on other metal-associated binding transporters has shown that cargo selection between the permease transmembrane domain (TMD) and the SBP occurs via a “scoop loop,” where hydrophilic residues in the permease interact with the cargo during the closed configuration in the SBP, accessing the cargo and initiating uptake through the permease (65, 66). Consequently, we performed a Clustal alignment between the permease FhuB from *Escherichia coli* and both permeases PiaB and PiaC in the pneumococcus to explore these residues (Fig. S10). The alignment revealed multiple conserved residues or residues with conserved properties, including methionine within the TMD. Many of these residues are implicated in resolving transport through the membrane and delivering cargo into the cytoplasm, but the substitutions potentially alter substrate interaction.

Together, our findings reveal a previously unrecognized route of copper entry in *S. pneumoniae* that becomes evident during simultaneous exposure to iron and copper. We propose that iron transporters functioning during iron transport facilitate copper uptake under mixed metal conditions through cooperative but spatially distinct interactions with the SBP. Copper transport would then depend on iron occupancy of the SBP, and mutations in key iron-coordinating residues may disrupt both iron and copper binding. Testing these possibilities will require targeted mutagenesis and biophysical characterizations, subject to further investigations.

Under physiological conditions, this mechanism remains unnoticed due to an efficient copper export system rapidly exporting copper out of the cell. Only by overwhelming this homeostatic system through synchronized Fe-Cu exposure does this mechanism become experimentally detectable. *In vivo,* we can hypothesize that this occurs during severe pneumococcal pneumonia. The convergence of tissue damage and inflammation can create one of the few host-niches where both iron and copper rise above their tightly regulated baselines (67). In the healthy lung, iron is sequestered by host proteins, limiting bacterial access, while copper is present at low levels (68). However, as infection progresses and inflammation increases, damage to the alveoli and endothelial barrier can lead to metal release (69–71). The release of blood cells into the alveolar space releases hemoglobin, which the pneumococcus degrades to acquire iron (72). This process circumvents host protein sequestration, transiently increasing the pool of available iron in the local niche.

Concurrently, inflammation recruits immune cells and increases vascular permeability, allowing copper-containing proteins such as ceruloplasmin to accumulate in the tissues (73, 74). As infection progresses, lysis of host cells contributes to metal release, including ferritin and hemoglobin-bound iron and intracellular copper, which would normally remain in compartments (68, 75, 76). This results in a dysregulated metal microenvironment in which both iron and copper are elevated. For the pneumococcus, this presents a challenge as iron is needed but can also catalyze redox reactions, while copper’s presence affects redox chemistry and mismetallation. This dual metal exposure would occur in localized regions where tissue destruction, vascular leakage, and immune infiltration occur. These conditions likely pressure pneumococcus to retain a robust metal homeostasis system capable of managing iron acquisition and copper detoxification, which become important during advanced stages of infection. These insights establish iron transport systems as relevant yet previously overlooked gateways for copper accumulation, reshaping our understanding of how metal homeostasis networks inversely facilitate copper acquisition.

Interestingly, we observed similar iron-enhanced copper increases in *S. aureus*, another gram-positive bacterium. Like pneumococcus, *S. aureus* has no known copper-requiring proteins; however, unlike pneumococcus, it produces siderophores and metallophores. Thus, iron-enhanced copper accumulation might represent a promiscuous copper entry mechanism operating in gram-positive bacteria that lack a dedicated copper import system but possess a characterized copper export system. These insights establish iron transport systems as relevant yet previously overlooked gateways for copper accumulation, reshaping our understanding of how metal homeostasis networks inversely facilitate copper acquisition.

## MATERIALS AND METHODS

### Bacterial growth

*S. pneumoniae* was routinely cultured overnight in tryptic soy agar (Criterion Tryptic Soy Agar C7122) plates supplemented with 5% sheep’s blood (QUAD FIVE, Ryegate MT) and incubated at 37°C, with 5% CO_2_. Starting inocula were taken directly from blood agar plates (BAP) or cultured overnight in RPMI_mod_. These bacteria were inoculated onto RPMI 1640 (Gibco 11835-030), which natively comes with 100 mg Ca(NO_3_)_2_ and 49 mg of MgSO_4_ per liter, with modifications (28) and supplemented with trace metals (246 µM MgCl_2_, 5.3 µM CaCl_2_, 179 nM FeSO_4_, 200 nM CuSO_4_, 173 nM ZnSO_4_, and 87 nM MnCl_2_) and 0.1 mg/mL of bovine liver catalase (Sigma C9322) (from here referred to as RPMI_mod_). Bacteria were grown to the mid-exponential phase (OD_600_ 0.3–0.4), and the culture was divided into the different treatments (200 µM copper, 200 µM iron, 200 µM copper, and 200 µM iron) and untreated. Metal solutions were prepared in Milli-Q water filtered to 18.1 MΩ. Iron solutions were prepared fresh before treatment to minimize the use of oxidized/insoluble iron. Incubation of treatments was at 37°C in a 5% CO_2_ incubator for 30 minutes. A separate set of experiments was performed switching Fe(NO_3_)_3_ · 9 H_2_O (Alfa Aesar 12226) for FeSO_4_ · 7 H_2_O (Sigma-Aldrich 215422). In some experiments, bacteria were also cultured on Todd Hewitt broth with 0.2% yeast extract.

### Inductively coupled plasma optical emission spectroscopy

A cold bath was made from a Styrofoam container filled with ice and salt and was used to rapidly cool down bacterial liquid samples to decrease metabolism, followed by centrifugation at 3500 × *g* at 4°C for 7 minutes and two washes of cold buffer (Tris 50 mM, NaCl 150 mM, EDTA 200 mM at pH 7.6). In total, 143 μL of trace metal grade HNO_3,_ at 70% (Macron 6623-46) was added to the total bacterial pellets, followed by overnight incubation at 65°C. After incubation, the lysed pellets were diluted to 2.5% HNO_3_ by adding 4.857 mL of ultra-pure water, which was filtered at 18.1 MΩ. As described below, bacterial plate counts were performed in TSA + 5% sheep’s blood through serial dilutions. Liquid samples were analyzed for metal content using an iCAP PRO XDUO ICP-OES with a wavelength of 324.754 nm for copper, 213.856 nm for zinc, 259.373 nm for manganese, and 259.940 nm for iron. Standards were prepared using IV-STOCK-27 ICP calibration standard (Inorganic Ventures), and metal content was calculated using the Qtegra software.

### Bacterial viability

Just before placing the liquid bacterial samples in the water bath, the conical tube containing the bacterial culture was shaken by hand three times to homogenize the sample. An amount of 200 µL of this culture was taken as a representative sample from each treatment and serially diluted in PBS. Bacterial counts were performed immediately on BAPs to quantify viable bacteria.

### Growth curves

Bacterial inocula were seeded onto a 96-well plate (Cellstar 655180 F-bottom) to an initial optical density of 0.05 and incubated at 37°C with 5% CO_2_ using RPMI_mod_. Measurements at 600 nm for optical density were taken every 30 minutes after a 5-second orbital shake in a Cytation 5.

### Gene knockouts

In-chromosomal deletion of the iron import systems, Sp_1032-1035 (*∆piaA-D*), Sp_1869-1872 (∆*piuA-D*), and Sp_1826-1824 (∆*afuA-C*), was performed by antibiotic resistance replacement using either erythromycin or spectinomycin or a combination as the selective markers, as described previously (77). Briefly, upstream and downstream fragments of the target gene were spliced to the antibiotic resistance cassette using the NEBuilder HiFi DNA Assembly master mix (New England Biolabs) following the manufacturer’s instructions and transformed into the bacteria and combined into the genome using homologous recombination. Bacteria were selected on blood agar plates with the respective antibiotic. DNA was extracted using the DNA extraction kit QIAamp DNA Mini kit purification (QIAGEN 51,304), and clones were sequence-verified (Eton Bio USA). The reference DNA sequence used was GenBank: AE005672.3. Table 1 contains the primer sequences used in this work.

**TABLE 1:** Primer sequences used to generate iron transport mutants

Name	Sequence
1869FW	CTGCTCTATCTTAGAAAAGGATAGTATTC
1869UPRV	GTAGATTTGGTATCGAAAGATATCTGC
1872RV	CGAAGTTGAAGGTCAACCTATC
1872DNFW	GCCACTTCAAACTCAATTTAATACTCAATG
ERMhybFW	ACCAAATCTACGGAAATAAGACTTAGAAGCAA
ERMhybRV	TTGAAGTGGCCCAAATTTACAAAAGCGAC
sp1032UPFW	GTTGGTTTCGTGTCATAACAGTTATAG
sp1032UPRV	AAAAACTCCTTAAACATATTTCAAGTCTATTG
sp1035DNFW	TAATTGAATATTGGAATTCATTTAAAAGTCGC
sp1035DNRV	GACTTAAAGTCTTTAATAGTGCCTTTCC
spechybfw	AAGGAGTTTTTatcgattttcgttcgtgaat
SPECHYBRV	CCAATATTCAATTAcatatgcaagggtttattg
1826upFW	CTTTAGAAAAAGTATCTGAAAAAACAGATG
1826upRV	GATAAGTTCTCCTTTTTTATTATTTTATTTAAATTTTTC
1824dwFW	TCATGACAGCCACTAGTCTTGG
1824dwRV	AAGAATGTTTCCAACTTCACGACAAATA
1826upspechyb	AAGGAGAACTTATCatcgattttcgttc
1824dwspechyb	TAGTGGCTGTCATGAcatatgcaag

### Iron-binding protein purification

#### Cloning and plasmid production

Target genes were amplified from *S. pneumoniae* TIGR4 genomic DNA using LIC primers designed with vector-specific overhangs. Amplicons were inserted into the pMCSG7 vector via ligation-independent cloning (LIC) as described by Stols et al. (78) (Table 2). The construct was transformed into *E. coli* XL-10 cells (Agilent) and plated on LB agar with 100 µg/mL ampicillin. Single colonies were cultured for plasmid extraction (QIAGEN plasmid mini kit) and Sanger sequencing (Eurofins). BL21-RIPL-competent cells (VWR) were transformed with 10 ng of the verified plasmid for protein expression.

**TABLE 2:** LIC vector primers

Primer name	Primer sequence
sp1872LICFw	**TACTTCCAATCCAAT**GCG**AGTACAAACTCAAGCACTAGTCAGAC**
sp1872LICRV	**TTATCCACTTCCAAT**GCGCTA**TTATTTCAAAGCTTTTTGTATGTCTTCAATCATG**
sp1032licFW	**TACTTCCAATCCAATGCGTCTTCTAATTCTGTTAAAAATGAAGAAAATACTTC**
sp1032LICRV	**TTATCCACTTCCAATGCGCTATTATTTCGCATTTTTGCATGCATTTCCTAAAAG**

#### LIC method

PCR primers contained 5′ overhangs matching the vector site, followed by gene-specific sequences. Amplified products were treated with T4 DNA polymerase and dCTP. The pMCSG7 vector was linearized with SspI-HF and treated with T4 DNA polymerase and dGTP to generate compatible overhangs.

#### Protein production

Transformed *E. coli* BL21-RIPL were streaked onto LB agar with 100 µg/mL ampicillin and incubated overnight at 37°C with 5% CO_2_. Colonies were resuspended in LB and used to inoculate 1 L of Terrific Broth (IBI Scientific) supplemented with 50 µg/mL ampicillin to an initial OD₆₀₀ of 0.03–0.05. Cultures were incubated at 37°C with 250 rpm shaking until mid-log phase (OD₆₀₀ = 0.6–0.8). Expression was induced with 500 µM IPTG after cooling the cultures below 30°C. Cultures were incubated overnight at 18°C with shaking. Bacterial pellets were harvested by centrifugation at 8,500 rpm for 30 minutes at 4°C and stored at −80°C.

#### Protein purification

Frozen pellets were thawed in buffer A (10 mL/g pellet). Lysis buffer was made by supplementing buffer A with 0.2 mg/mL DNase I (GoldBio), 1 mM PMSF, 20 µM leupeptin hemisulfate, and 14 µM pepstatin A (GoldBio). Cells were lysed by sonication on ice, and lysates were clarified by centrifugation at 18,500 rpm for 30 minutes at 4°C. Supernatants were applied to a HisTrap Crude FF column (Cytiva) and eluted with buffer B. Fractions containing protein were pooled and run on the HiPrep 26/10 Desalting column (Cytiva) into Base Buffer. The eluted protein was incubated overnight at 4°C with TEV protease and 1 mM DTT. The following day, samples were passed again over the HisTrap column for final purification. Buffer A contains 50 mM Tris, 150 mM NaCl, 5% glycerol, and 25 mM imidazole, pH 7.6. Buffer B contains 50 mM Tris, 150 mM NaCl, 5% glycerol, and 250 mM imidazole, pH 7.6). Base buffer contains 50 mM Tris, 150 mM NaCl, and 5% glycerol, pH 7.6.

#### Metal treatment

Purified protein was divided into treatment groups: Fe²^+^ [iron(II) sulfate heptahydrate], Fe³^+^ [iron(III) nitrate nonahydrate], Cu²^+^ [copper(II) sulfate pentahydrate], Fe²^+^ + Cu²^+^ combined, or Fe³^+^ + Cu²^+^ combined. All metal stocks were prepared in TBS buffer. Proteins were incubated at room temperature for 10 minutes and then purified on the HiPrep 26/10 Desalting column (Cytiva) with TBS buffer. Between samples, the system was washed with 1 mM EDTA to prevent metal cross-contamination. Eluted proteins were digested overnight at 65°C in 70% nitric acid prior to ICP-OES analysis.

Iron II (iron II sulfate heptahydrate. ACS grade) was from Sigma-Aldrich (lot MKCF5205, Fw 278.01). Iron III (iron III nitrate nonahydrate >98% metals basis) was from Alfa Aesar (lot R16D018, Fw 403.99). Copper II (cupric sulfate, pentahydrate, ACS grade) was from Caisson Lab (lot 09172024, Fw 249.69).

#### ICP-OES analysis

Nitric acid-digested samples were diluted in 2% HNO₃ for analysis. Custom standards (Inorganic Ventures) were diluted to 0.1, 0.5, and 1.0 ppm using MilliQ water and used to generate a calibration curve. Metal content was analyzed using an iCAP Pro X Duo ICP-OES system (Thermo Fisher). The sample uptake was 40 seconds with five replicates and a 40-second rinse between samples. The emission lines used for analysis were as follows: copper, 324.754 nm; iron, 259.940 nm; manganese, 259.373 nm; zinc, 213.856 nm.

### Transcriptome analysis

#### Sample collection and preparation

Bacteria were grown in RPMI_mod_ to mid-exponential phase (0.3–0.4 OD 600 nm), then exposed to 200 µM CuSO_4_, 200 µM FeSO_4_, a combination of both metals, or an untreated control. A parallel culture was grown with 100 µM FeSO_4_ for approximately 300 minutes, to the mid-exponential phase, then centrifuged and resuspended in fresh medium containing 200 µM CuSO_4_ and 200 µM FeSO_4_. All cultures were incubated for 30 minutes with the metal conditions under 5% CO_2_ at 37°C. RNA was stabilized by using a 1:2 ratio of bacteria culture to Bacterial RNA protect reagent (Qiagen) following the manufacturer’s instructions, followed by 100 µL of lysozyme (15 mg/mL) solution and 20 µL of proteinase K (20 mg/mL). The pellet was stored at −80°C, then sent to Novogene for transcriptome sequencing and analysis.

#### RNA quantification and qualification

RNA degradation and contamination were monitored on 1% agarose gels.

RNA purity was checked using the Nanophotometer spectrophotometer (IMPLEN, CA, USA)

RNA integrity and quantitation were assessed using the RNA Nano 6000 Assay kit of the Bioanalyzer 2100 system (Agilent Technologies, CA, USA)

#### Library preparation for transcriptome sequencing

A total amount of 1 μg RNA per sample was used as input material for the RNA sample preparations. Sequencing libraries were generated using NEBNext Ultra RNA Library Prep Kit for Illumina (NEB, USA) following the manufacturer’s recommendations, and index codes were added to attribute sequences to each sample. Briefly, mRNA was purified from total RNA using poly-T oligo-attached magnetic beads. Fragmentation was carried out using divalent cations under elevated temperature in NEBNext First-Strand Synthesis Reaction Buffer (5×). First-strand cDNA was synthesized using random hexamer primers and M-MuLV reverse transcriptase (RNase H). Second-strand cDNA synthesis was subsequently performed using DNA polymerase I and RNase H. Remaining overhangs were converted into blunt ends via exonuclease/polymerase activities. After adenylation of 3′ ends of DNA fragments, NEBNext Adaptor with hairpin loop structure was ligated to prepare for hybridization. To select cDNA fragments of preferentially 150–200 bp in length, the library fragments were purified with the AMPure XP system (Beckman Coulter, Beverly, USA). Then 3 μL USER Enzyme (NEB, USA) was used with size-selected, adaptor-ligated cDNA at 37°C for 16 minutes, followed by 5 minutes at 95°C before PCR. Then, PCR was performed with Phusion High-Fidelity DNA polymerase, Universal PCR primers, and Index (X) Primer. At last, PCR products were purified (AMPure XP system), and library quality was assessed on the Agilent Bioanalyzer 2100 system.

#### Clustering and sequencing (Novogene Experimental Department)

The clustering of the index-coded samples was performed on a cBot Cluster Generation System using PE Cluster Kit cBot-HS (Illumina) according to the manufacturer’s instructions. After cluster generation, the library preparations were sequenced on an Illumina platform, and 125 bp/150 bp paired-end reads were generated.

### Data analysis

#### Quality control

Raw data (raw reads) of fastq format were first processed. In this step, clean data (clean reads) were obtained by removing reads containing adapters, reads containing poly-N, and low-quality reads from the raw data. At the same time, Q20, Q30, and GC content of the clean data were calculated. All the downstream analyses were based on the clean data with high quality.

#### Reads mapping to the reference genome

Reference genome and gene model annotation files were downloaded from the genome website directly. An index of the reference genome was built using hisat2 2.1.0, and paired-end clean reads were aligned to the reference genome using HISAT2. We selected HISAT2 as the mapping tool because Hisat2 can generate a database of splice junctions based on the gene model annotation file, and thus provides a better mapping result than other non-splice mapping tools, which is a fast and sensitive alignment program for mapping next-generation sequencing reads against the general human population.

#### Quantification of gene expression level

FeatureCounts v1.5.0-p3 was used to count the read numbers mapped to each gene (79). And then FPKM of each gene was calculated based on the length of the gene and the read count mapped to this gene. FPKM, expected number of fragments per kilobase of transcript sequence per million base pairs sequenced, considers the effect of sequencing depth and gene length for the read count at the same time, and is currently the most commonly used method for estimating gene expression levels (80).

#### Differential expression analysis

For DESeq2 with biological replicates, differential expression analysis between two conditions/groups (two biological replicates per condition) was performed using the DESeq2 R package (1.14.1). DESeq2 provides a statistical routine for determining differential expression in digital gene expression data using a model based on the negative binomial distribution. The resulting *P*-values were adjusted using Benjamini and Hochberg’s approach for controlling false discovery rate (FDR), with an adjusted *P*-value <0.05 found by DESeq2, were assigned as differentially expressed.

For edgeR without biological replicates, prior to differential gene expression analysis, for each sequenced library, the read counts were adjusted by the edgeR program package through one scaling normalization factor. Differential expression analysis of two conditions was performed using the edR R package (3.16.5). The *P* values were adjusted using the Benjamini and Hochberg method. Corrected *P*-values of 0.05 and an absolute fold change of 1 were set as the threshold for significantly differential expression.

#### GO and KEGG enrichment analysis of differentially expressed genes

Gene ontology (GO) enrichment analysis of differentially expressed genes was implemented by the clusterProfiler R package, in which gene length bias was corrected. GO terms with corrected *P* value less than 0.05 were considered significantly enriched by differentially expressed genes.

KEGG is a database resource for understanding high-level functions and utilities of the biological system, such as the cell, the organism, and the ecosystem, from molecular-level information, especially large-scale molecular data sets generated by genome sequencing and other high-throughput experimental technologies (https://www.genome.jp/kegg/). We used the clusterProfiler R package to test the statistical enrichment of differentially expressed genes in KEGG pathways.

#### Protein-protein interaction analysis of differentially expressed genes

Protein-protein interaction (PPI) analysis of differentially expressed genes was based on the STRING database, which has known and predicted protein-protein interactions. For the species existing in the database, we constructed the networks by extracting the target gene list from the database. Otherwise, Blastx (v2.2.28) was used to align the target gene sequences to the selected reference protein sequences, and then the networks were built according to the known interactions of the selected reference species.

#### Novel transcripts prediction and alternative splicing analysis

The Cufflinks v2.1.1 Reference Annotation Based Transcript (RABT) assembly method was used to construct and identify both known and novel transcripts from TopHat alignment results. Alternative splicing events were classified into 12 basic types by the software Asprofile v1.0. The number of AS events in each sample was estimated separately.

#### SNP analysis

Picard tools (v1.111) and samtools v0.1.18 were used to sort, mark duplicated reads, and reorder the bam alignment results of each sample. GATK4.1 software was used to perform SNP calling.

### Proteome analysis

#### Sample preparation for mass spectrometry analysis

For sample denaturation, reduction, and alkylation, frozen cell pellets were suspended in 2 mL of 1 × PBS, pH 7.49 (Gibco, CAT# 10010023), centrifuged for 5 minutes at 7,000 × *g*, room temperature, and supernatants were removed. Samples were suspended in 700 µL of lysis buffer composed of 6M urea (Fisher Chemicals, CAT# U15-500), 7% SDS (Fisher BioReagents, CAT# BP166-500), 50 mM triethylammonium bicarbonate (TEAB) (Sigma-Aldrich, CAT# T7408-500ML), pH 7.7; supplemented with Thermo Scientific HALT Protease and Phosphatase Inhibitor Cocktail, EDTA-free (100×) (Fisher Scientific, CAT# PI78445) at a final concentration of 1×; transferred into 2 mL screw cap tubes (Sarstedt, CAT# 72.693.005) containing 2.3 mm zirconia/silica beads (BioSpec Products, CAT# 11079125Z) and subjected to five rounds of 1 minute bead beating at 4°C in a Mini-Beadbeater-24 (BioSpec Products, CAT# 112011) with 1 minute of rest between cycles. Cell suspensions were next transferred into 2 mL Protein LoBind Tubes (Eppendorf, CAT# 022431102), sonicated using Qsonica Sonicator Q125 (Qsonica LLC, CAT# Q125-110) equipped with a 1.6 mm microtip probe (Qsonica LLC, CAT# 4417) at the following setting: amplitude 20, 3-second pulse, 5-second break, five pulses; and finally, sonicated for 5 minutes in a Branson 2800 Ultrasonic Bath (Branson Ultrasonics, CAT# CPX952219R). Lysates were centrifuged for 5 minutes at 16,100 × *g*, at room temperature, and 500 µL of the supernatants was transferred into a 2 mL deep well plate (Eppendorf, CAT# 30504305). Proteins were chemically reduced by the addition of 5 µL of 0.5 M dithiothreitol (DTT) (Invitrogen, CAT# 15508-013) and a 30-minute incubation at 47°C. Next, samples were cooled on ice for 5 minutes, and proteins were alkylated by the addition of 15 µL of 0.5 M iodoacetamide (Sigma-Aldrich, CAT# I1149) and a 45-minute incubation at room temperature in the dark. The alkylation reaction was stopped by adding 5 µL of 0.5 M DTT and incubating for 15 minutes at room temperature.

#### Protein digestion

Samples were split in half on the 2 mL deep well plate, acidified by the addition of 27 µL of 12% phosphoric acid (Sigma-Aldrich, CAT# 49685-500ML), mixed with 1.5 mL of binding buffer composed of 90% methanol (Fisher Chemicals, CAT# A456-4), 50 mM TEAB, pH 7.7, and loaded, ~350 µL at a time, onto a 96-well S-Trap plate (ProtiFi, CAT# C02–96well-10), placed on a 1.1 mL deep well plate (Axygene, CAT# P-DW-11-C), by 1- to 2-minute centrifugation at 1,500 × *g*, room temperature. Wells were washed five times with 200 µL of binding buffer by 1-minute centrifugation at 1,500 × *g* at room temperature. The S-trap plate was next centrifuged for 5 minutes at 1,500 × *g*, at room temperature, to remove residual methanol, and then transferred onto new 1.1 mL deep well plates. Trypsin digestion mix, composed of 20 µL (10 µg) of Sequencing Grade Modified Trypsin (Promega, CAT# V5113) and 105 µL of 50 mM TEAB, was loaded into each well, the plate was briefly centrifuged (~4 seconds at <1,500 × *g*, room temperature), resulting flow-through was reapplied onto the wells, and the plate was incubated for 3 hours at 47˚C. Peptides were eluted from the wells with 125 µL of 50 mM TEAB (1-minute centrifugation at 2,000 × *g*, room temperature), 125 µL of 5% formic acid (Fisher Chemicals, CAT# A117-50) (1-minute centrifugation at 2,000 × *g*, room temperature), and 125 µL of 50% acetonitrile (Fisher Chemicals, CAT# A955-500) (3-minute centrifugation at 2,000 × *g*, room temperature). S-Trap plate was discarded, 1.1 mL deep well plates containing eluted peptides were covered with a silicone sealing mat (Axygen, CAT# AM-2ML-RD), and the samples were transferred to −80℃. Frozen peptides were dried using Savant SPD111V SpeedVac Concentrator (Thermo Scientific, CAT# SPD111V-115) in line with Savant RVT5105 Refrigerated Vapor Trap (Thermo Scientific, CAT# RVT5105-115).

#### Peptide desalting

Dried peptides were suspended in 500 µL of 0.1% trifluoroacetic acid (TFA) (Thermo Fisher Scientific, CAT# 28901) and incubated for 20 minutes in a MixMate Mixer (Eppendorf, CAT# 5353) at 1,000 rpm, room temperature. Sep-Pak C18 96-well plate (100 mg sorbent) (Waters, CAT# WAT-186002321), placed on a 1.1 mL deep well plate, was washed with 1 mL of 100% acetonitrile and twice with 1 mL of 0.1% TFA by 1-minute centrifugation at 300 × *g*, room temperature. Samples were applied onto the wells and centrifuged for 1 minute at 300 × *g*, room temperature. Wells were washed five times with 1 mL of 0.1% TFA by 1-minute centrifugation at 300 × *g*, room temperature, and a Sep-Pak C18 96-well plate was transferred onto a new 1.1 mL deep well plate. Peptides were eluted with 750 µL of 40% acetonitrile, 0.5% acetic acid (Fisher Chemicals, CAT# A35-500) and 750 µL of 80% acetonitrile, 0.5% acetic acid by 1-minute centrifugation at 300 × *g*, room temperature. Eluted peptides were transferred into a 2 mL deep well plate, covered with a silicone sealing mat (Axygen, CAT# AM-2ML-SQ), and transferred to 80℃. Frozen samples were dried using a Savant SPD111V SpeedVac Concentrator in line with a Savant RVT5105 Refrigerated Vapor Trap.

#### Peptide quantification

Dried peptides were suspended in 1 mL of 50% acetonitrile and incubated for 20 minutes in a MixMate Mixer at 1,000 rpm, room temperature. Pierce Quantitative Colorimetric Peptide Assay kit (Thermo Fisher Scientific, CAT# 23275) was used according to the manufacturer’s recommendations to determine peptide concentration. Transferred 20 µg aliquots of each sample into 1.5 mL Protein LoBind Tubes, frozen at −80℃, and dried using Savant SPD111V SpeedVac Concentrator in line with Savant RVT5105 Refrigerated Vapor Trap.

For sample reconstitution for mass-spectrometry analysis, dried samples were suspended in 40 µL of 5% acetonitrile and 5% formic acid and mixed for 20 minutes in a Vortex-Genie 2 vortex (Scientific Industries, CAT# 00-SI-0236), sonicated for 5 minutes in a Branson 2800 Ultrasonic Bath, and centrifuged for 2 minutes at 16,100 × *g*, room temperature. Supernatants were transferred into SureSTART 0.2 mL Amber TPX vials with Glass Insert (Thermo Scientific, CAT# 6PK1655).

#### Mass spectrometry data acquisition

Proteomic spectral data were collected using a data-independent acquisition (DIA) approach with a Thermo Scientific Orbitrap Astral Mass Spectrometer. A 0.6 μL (300 ng) of each sample was separated by reverse-phase high pH liquid chromatography using Vanquish Neo UHPLC System (Thermo Fisher Scientific) and Aurora Elite XT 15 × 75 C18 UHPLC column (IonOpticks, CAT# AUR4-15075C18-XT). Solvent A of the mobile phase consisted of 0.1% formic acid in water, while solvent B consisted of 0.1% formic acid in 80% acetonitrile. Samples were separated by a 25-minute method composed of a linear gradient of 1%–4% solvent B at minute 0–0.1 with a flow rate of 700 nL/min, a linear gradient of 4%–12% solvent B at minute 0.1–1.9 with a flow rate of 700 nL/min, a linear gradient of 12%–22.5% solvent B at minute 2–12 with a flow rate of 700 nL/min, a linear gradient of 22.5%–40% solvent B at minute 12–19.5 with a flow rate of 500 nL/min, a linear gradient of 40%–99% solvent B at minute 19.5–22 with a flow rate of 500 nL/min, and an isocratic flow of 99% solvent B at minute 22–25 with a flow rate of 600 nL/min.

Data were acquired with the following settings: experiment #1 (MS) master scan using full scan at Orbitrap Resolution 240,000, scan range of 380–980 *m*/*z*, RF lens 40%, custom AGC target, normalized AGC target 500%, absolute AGC value 5.000e6, maximum injection time 3 ms, microscans set to 1, data type set to profile, positive polarity; experiment #1 (DIA) master scan using DIA at precursor mass range 380980, DIA window type set to auto, isolation window 2 *m*/*z*, wwindow overlap set to 0 *m*/*z*, window placement optimization turned on, number of scan events set to 299, DIA window mode set to *m*/*z* range, normalized collision energy type, HCD collision energy set to 25%, astral detector type, scan range of 150–2,000 *m*/*z*, RF lens 40%, custom AGC target, normalized AGC target 500%, absolute AGC value 5.000e4, maximum injection time 3 ms, microscans set to 1, data type set to centroid, positive polarity, loop control set to time with 0.6 s.

#### Mass spectrometry data search

For the DIA analysis, an *in* silico predicted spectral library was generated from a UniProt reference proteome of *Streptococcus pneumoniae* TIGR4 (accession UP000000585), using DIA-NN software (version 2.0 Academia) with the following settings: FASTA digest for library-free search/library generation; deep learning-based spectra, RTs and IMs prediction; protease = trypsin/P; missed cleavages = 1, maximum number of variable modifications = 1, and modifications = N term M excision, C carbamidomethylation. Thermo Finnigan RAW spectra files with DIA data were imported into the DIA-NN software and searched against the generated spectral library using the following settings: protease = trypsin/P; missed cleavages = 1; maximum number of variable modifications = 1; modifications = N term M excision, C carbamidomethylation, Ox(M); use isotopologues; MBR; heuristic protein inference, no shared spectra; protein interference = genes; neural network classifier = single pass mode; quantification strategy = robust LC (high precision); cross-run normalization = RT-dependent; library generation = smart profiling; precursor FDR (%) =1.0; mass accuracy, MS1 accuracy, and scan window were used at the default (0) setting.

#### Mass spectrometry data statistical analysis

FragPipe-Analyst online tool (https://fragpipe-analyst.org/) was used to perform all statistical analyses on the obtained proteomics data.

### Statistics, figures, and graphics

Statistical analysis and figures were prepared using the software GraphPad Prism 10. Graphics were created using the free version of Biorender.

### Sequence Alignment

Protein sequences were obtained from the Uniprot database and the web server Clustal Omega was used for sequence alignments.

## Data Availability

All proteomics data (.raw files and metadata) are publically available online at MassIVE Repository (https://massive.ucsd.edu) under identifier MSV000102036.

## SUPPLEMENTAL MATERIAL

Supplemental Figures msphere.00165-26-s0001.pdf Figures S1 to S10.

Legends msphere.00165-26-s0002.docx Supplemental figure legends.

Table S1 msphere.00165-26-s0003.xlsx RNAseq data.

Table S2 msphere.00165-26-s0004.xlsx Proteomics data set.

ASM does not own the copyrights to Supplemental Material that may be linked to, or accessed through, an article. The authors have granted ASM a non-exclusive, world-wide license to publish the Supplemental Material files. Please contact the corresponding author directly for reuse.
