# Supplementary material for: Iron and its import systems enhance copper accumulation in Streptococcus pneumoniae
Source: mSphere. 2026 Jun 10;11(6):e00165-26. doi: 10.1128/msphere.00165-26 (PMC13317198; doi:10.1128/msphere.00165-26)
Supplement: Supplemental Figures — Figures S1 to S10. [file msphere.00165-26-s0001.pdf]

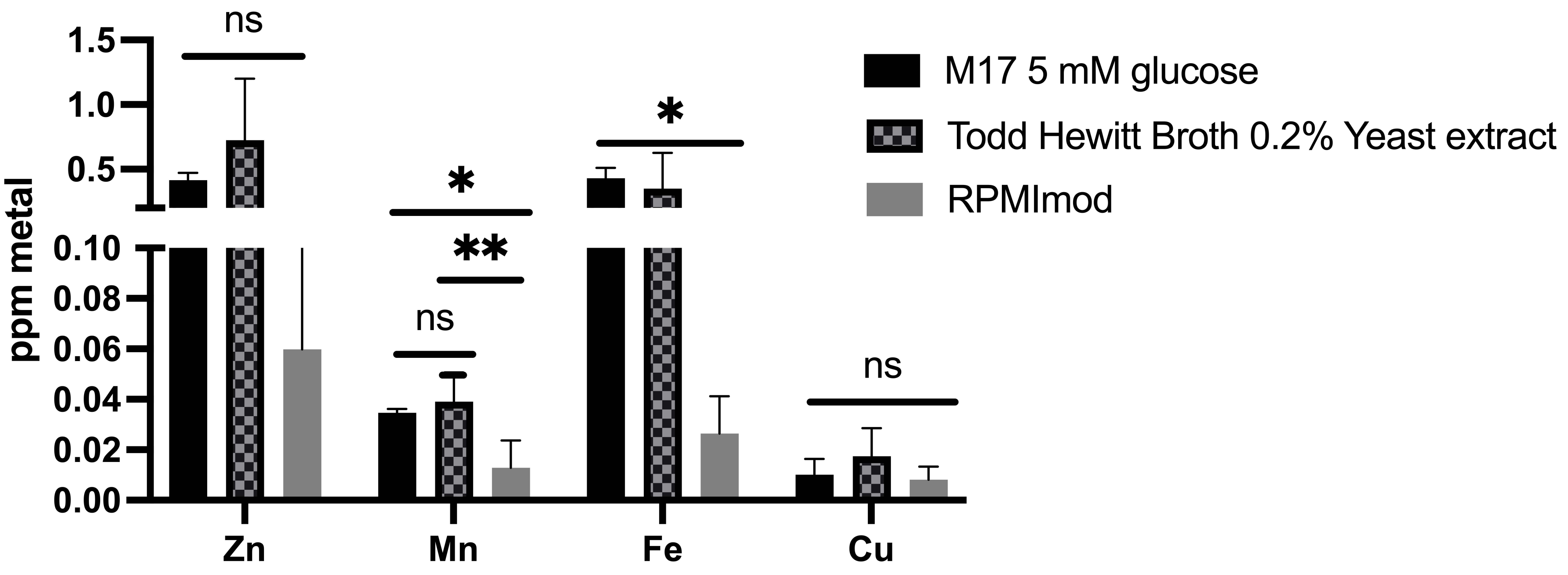

ns
1.5
1.0
0.5
*
*
**
ns
ns
0.10
0.08
0.06
0.04
0.02
0.00
ppm metal
Zn
Mn
Fe
Cu
M17 5 mM glucose
Todd Hewitt Broth 0.2% Yeast extract
RPMImod

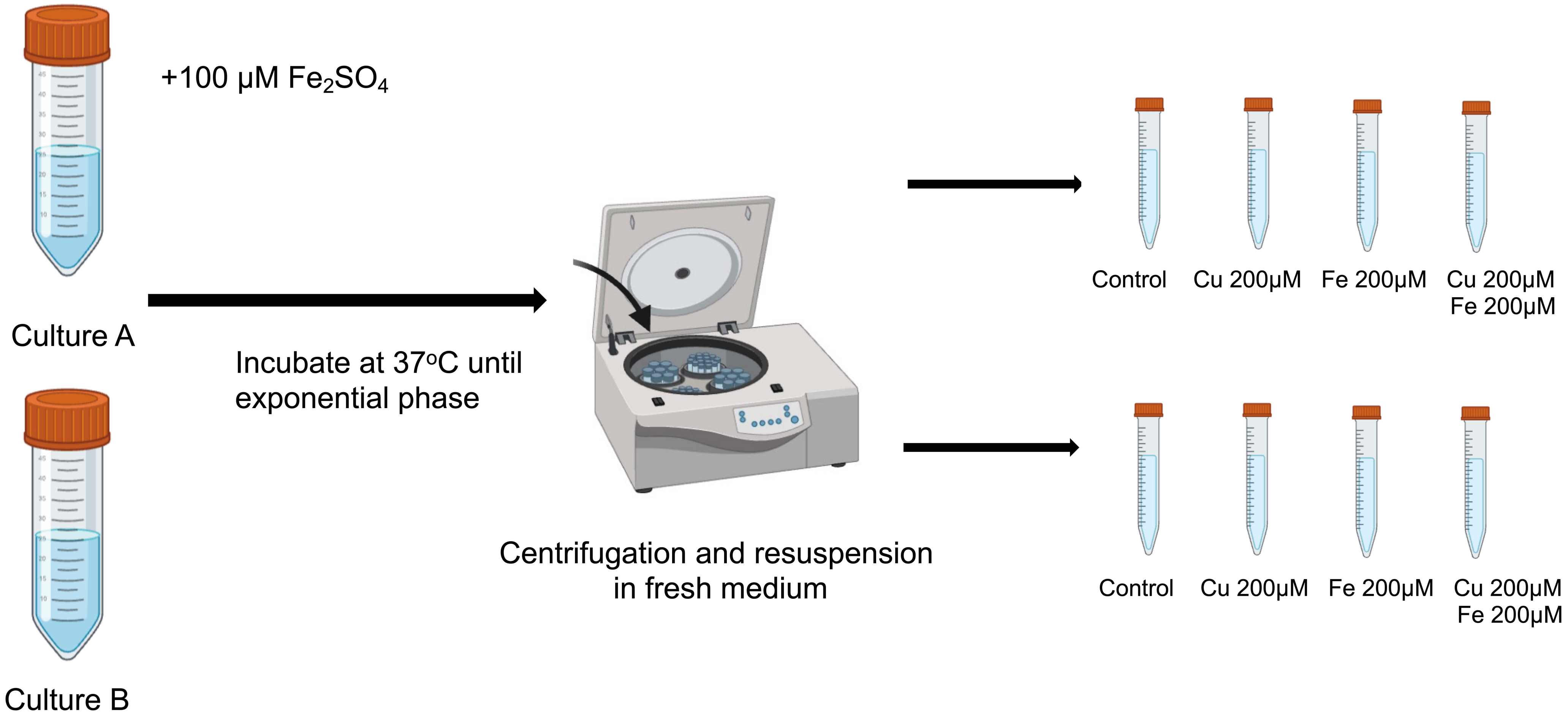
+100 µM $Fe_2SO_4$
Culture A
Culture B
Incubate at 37$^{o}$C until exponential phase
Centrifugation and resuspension in fresh medium
Control
Cu 200µM
Fe 200µM
Cu 200µM Fe 200µM
Control
Cu 200µM
Fe 200µM
Cu 200µM Fe 200µM

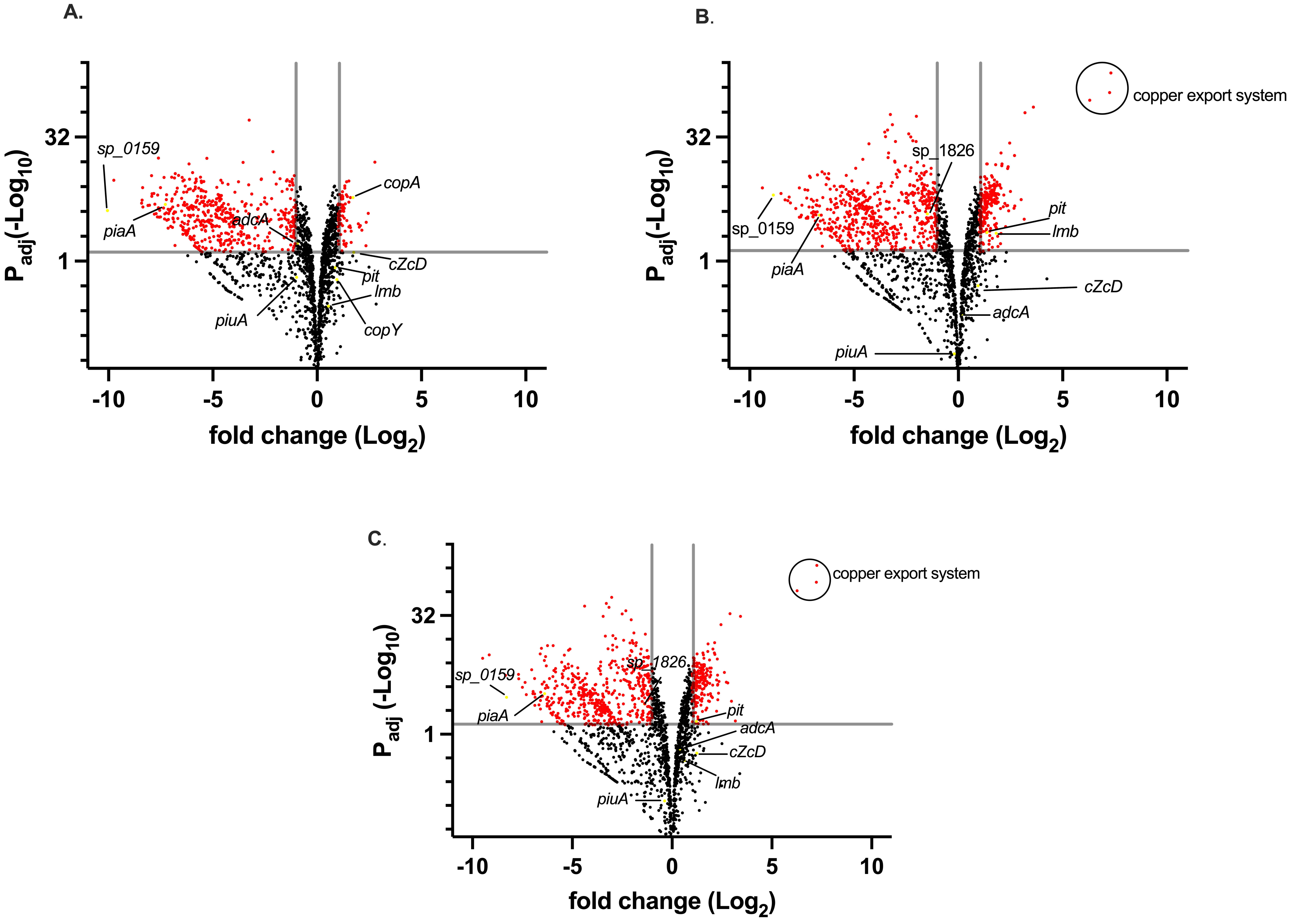
A.
B.
C.
copper export system
copper export system
32
1
32
1
32
1
Padj(-Log10)
Padj(-Log10)
Padj (-Log10)
-10
-5
0
5
10
fold change (Log2)
fold change (Log2)
fold change (Log2)
sp_0159
piaA
adcA
copA
cZcD
pit
lmb
copY
piuA
sp_1826
sp_0159
piaA
pit
lmb
cZcD
adcA
piuA
sp_0159
piaA
sp_1826
pit
adcA
cZcD
lmb
piuA

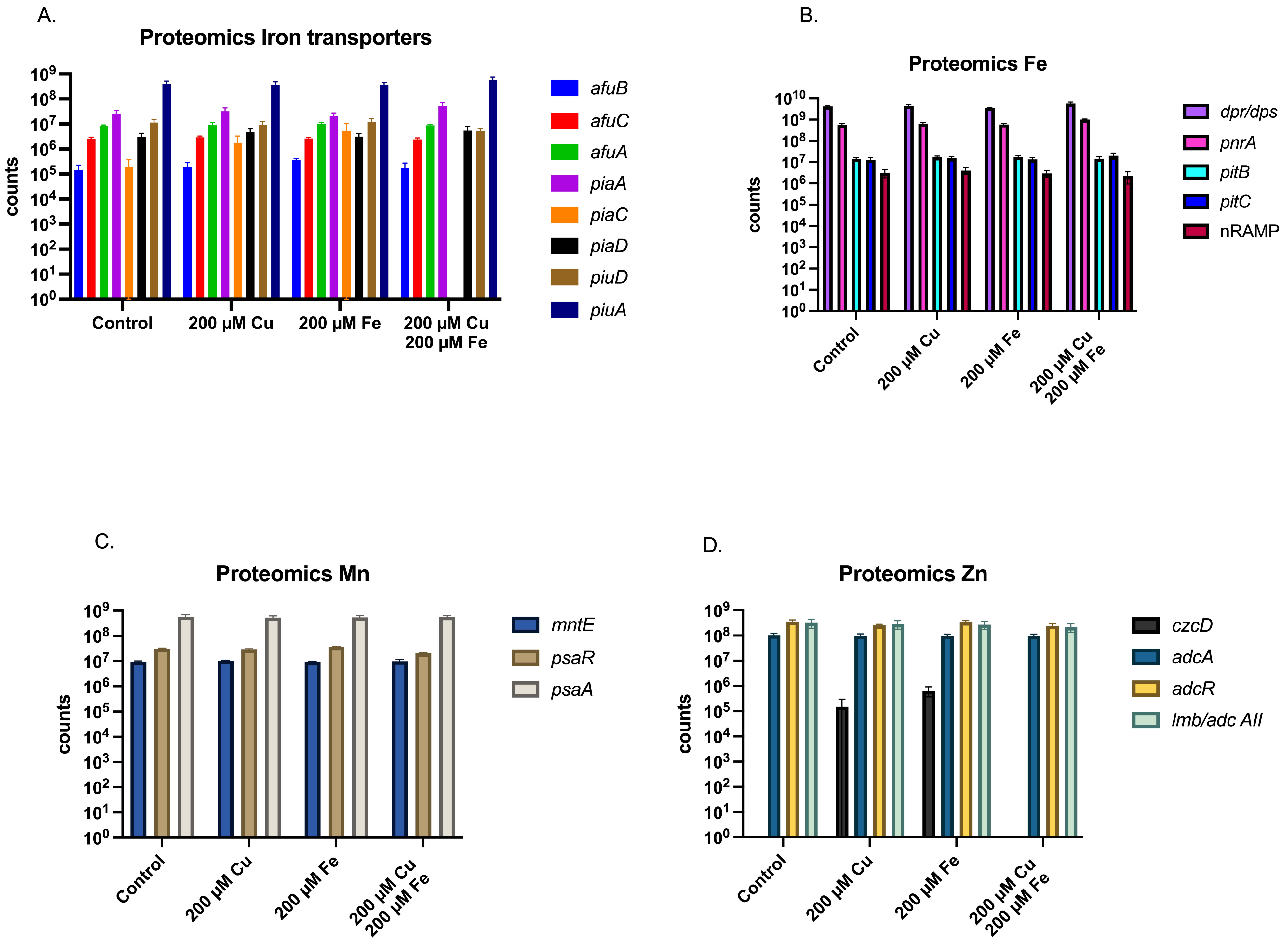
A.
Proteomics Iron transporters
counts
Control
200 µM Cu
200 µM Fe
200 µM Cu
200 µM Fe
afuB
afuC
afuA
piaA
piaC
piaD
piuD
piuA
B.
Proteomics Fe
counts
Control
200 µM Cu
200 µM Fe
200 µM Cu
200 µM Fe
dpr/dps
pnrA
pitB
pitC
nRAMP
C.
Proteomics Mn
counts
Control
200 µM Cu
200 µM Fe
200 µM Cu
200 µM Fe
mntE
psaR
psaA
D.
Proteomics Zn
counts
Control
200 µM Cu
200 µM Fe
200 µM Cu
200 µM Fe
czcD
adcA
adcR
lmb/adc AII

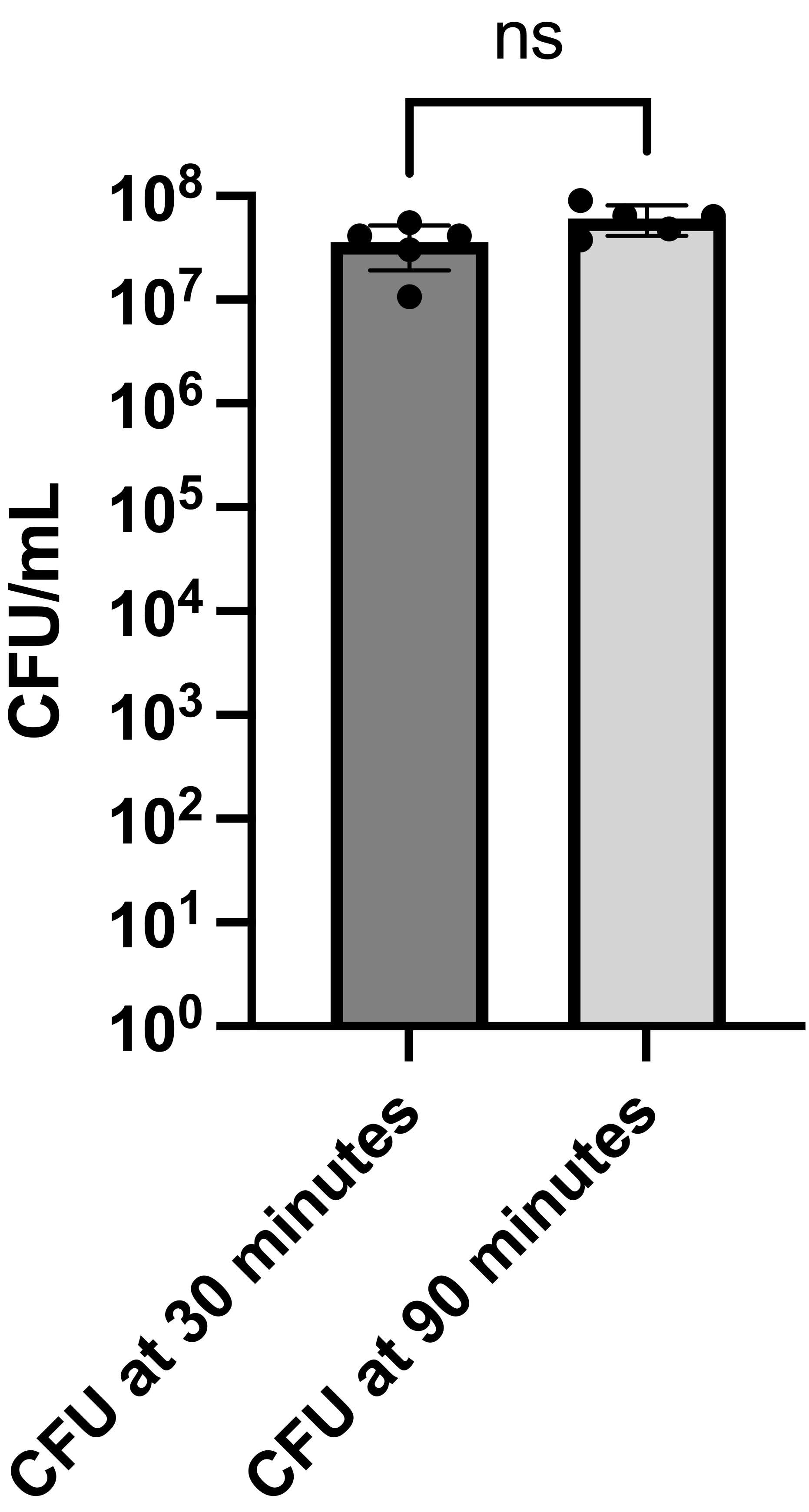

ns
CFU/mL
$10^8$
$10^7$
$10^6$
$10^5$
$10^4$
$10^3$
$10^2$
$10^1$
$10^0$
CFU at 30 minutes
CFU at 90 minutes

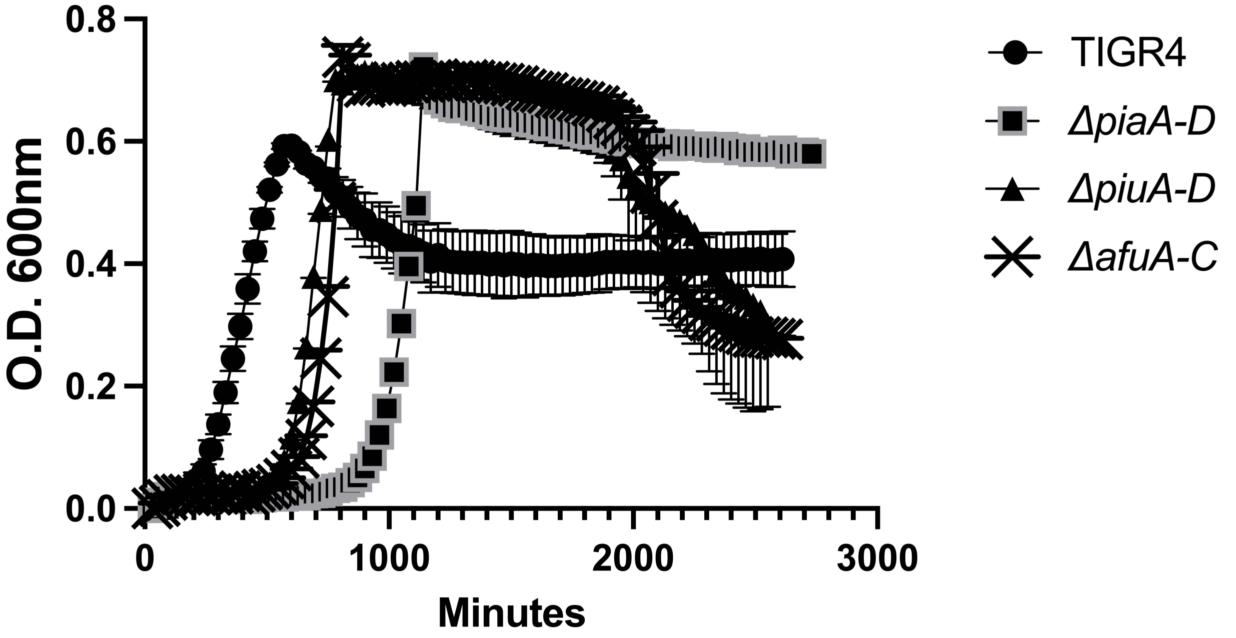

O.D. 600nm
0.0
0.2
0.4
0.6
0.8
0
1000
2000
3000
Minutes
TIGR4
ΔpiaA-D
ΔpiuA-D
ΔafuA-C

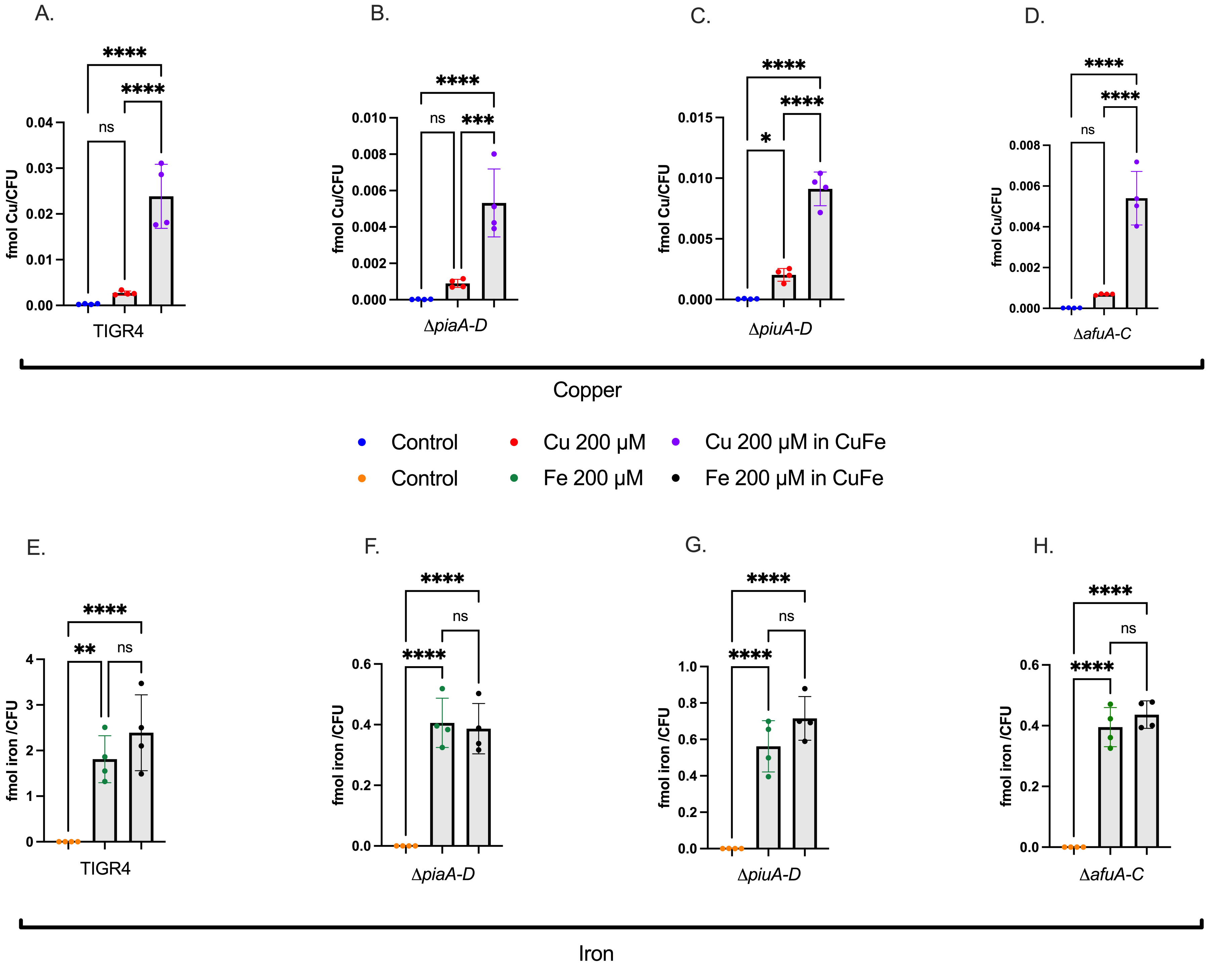

A.
B.
C.
D.
fmol Cu/CFU
TIGR4
ΔpiaA-D
ΔpiuA-D
ΔafuA-C
ns
*
***
****
Copper
Control
Cu 200 μM
Cu 200 μM in CuFe
Control
Fe 200 μM
Fe 200 μM in CuFe
E.
F.
G.
H.
fmol iron /CFU
**
Iron

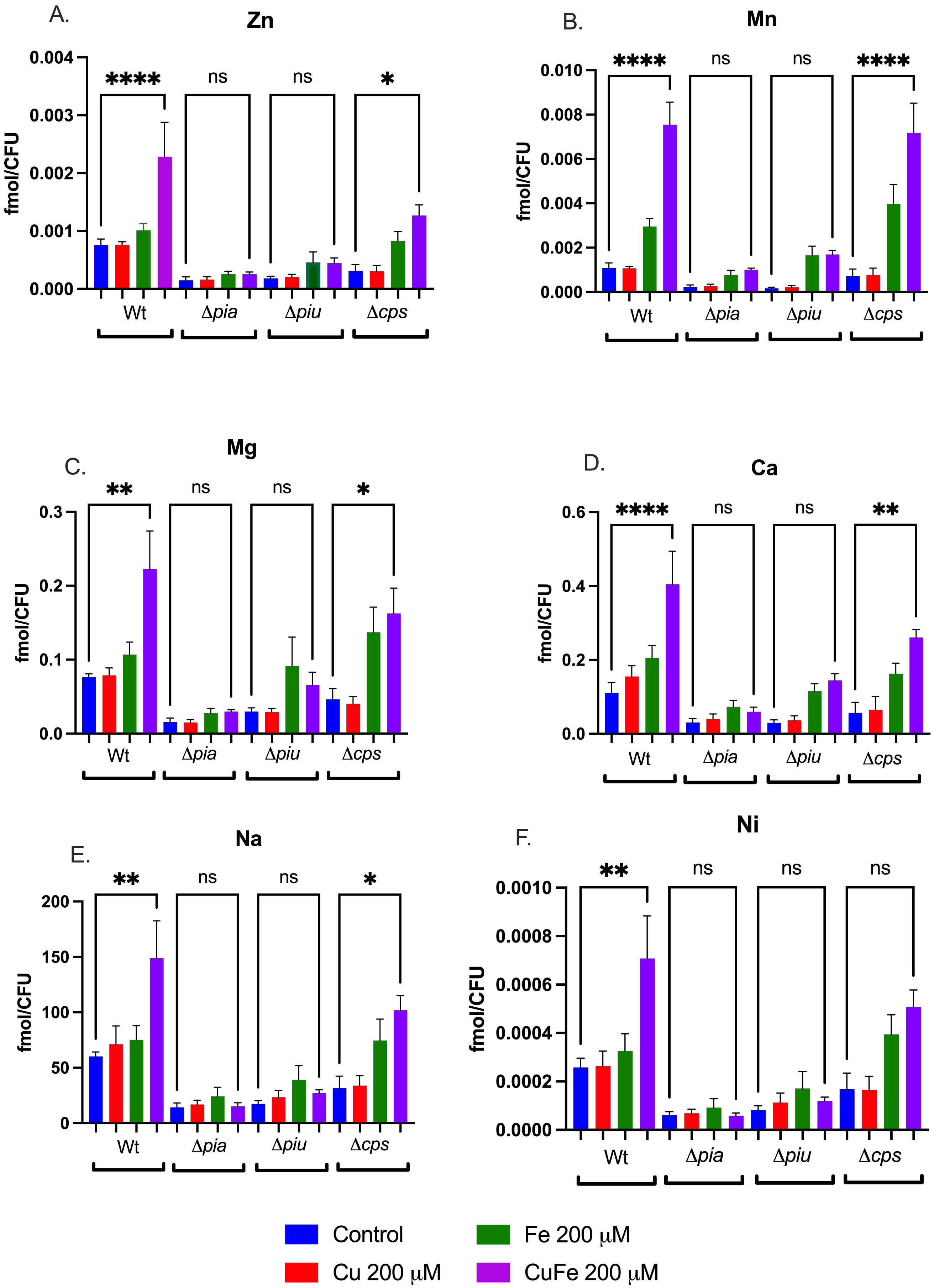
A.
Zn
fmol/CFU
0.004
0.003
0.002
0.001
0.000
****
ns
ns
*
Wt
Δpia
Δpiu
Δcps
B.
Mn
fmol/CFU
0.010
0.008
0.006
0.004
0.002
0.000
****
ns
ns
****
Wt
Δpia
Δpiu
Δcps
C.
Mg
fmol/CFU
0.3
0.2
0.1
0.0
**
ns
ns
*
Wt
Δpia
Δpiu
Δcps
D.
Ca
fmol/CFU
0.6
0.4
0.2
0.0
****
ns
ns
**
Wt
Δpia
Δpiu
Δcps
E.
Na
fmol/CFU
200
150
100
50
0
**
ns
ns
*
Wt
Δpia
Δpiu
Δcps
F.
Ni
fmol/CFU
0.0010
0.0008
0.0006
0.0004
0.0002
0.0000
**
ns
ns
ns
Wt
Δpia
Δpiu
Δcps
Control
Cu 200 μM
Fe 200 μM
CuFe 200 μM

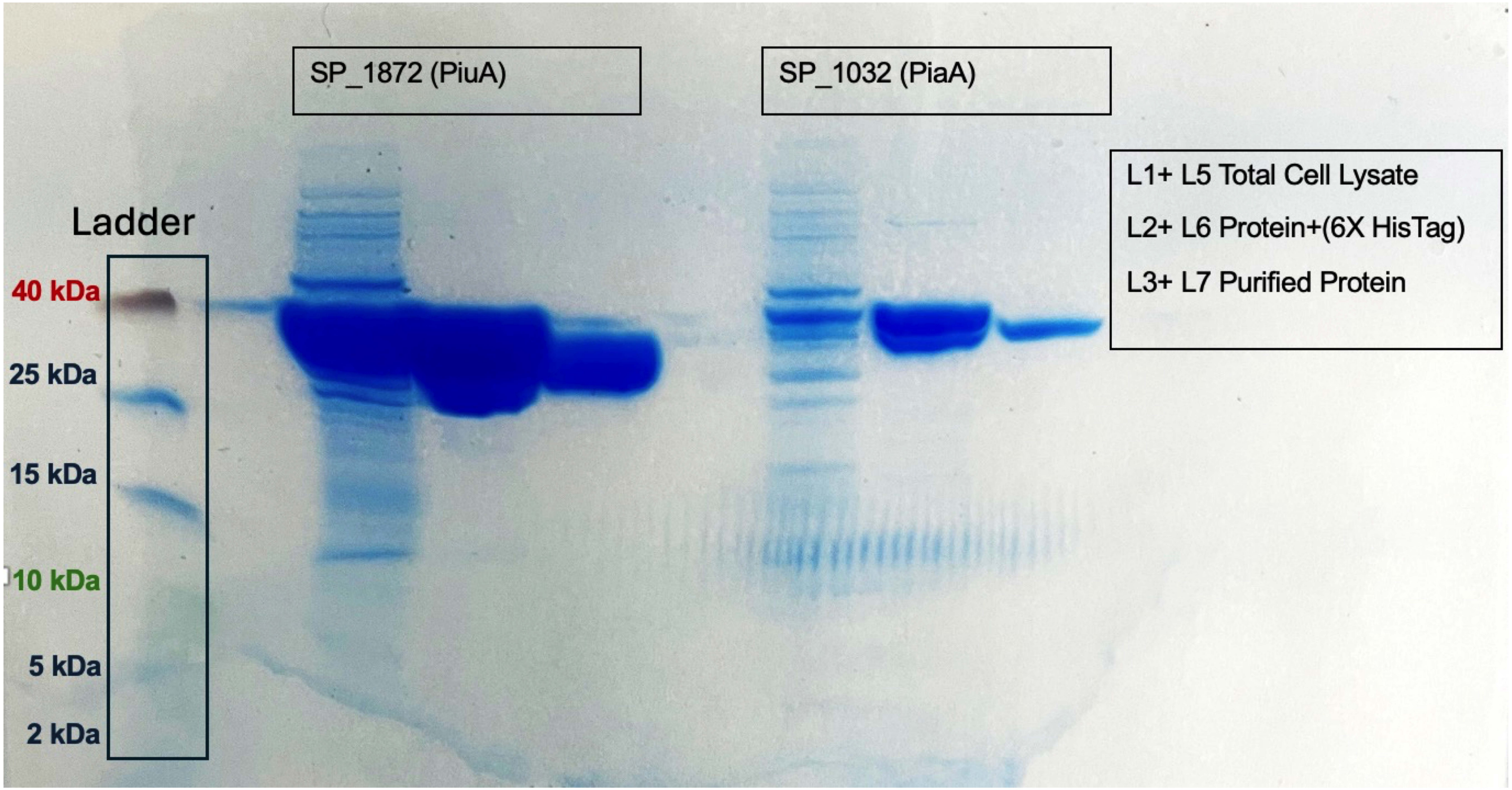
SP_1872 (PiuA)
SP_1032 (PiaA)
Ladder
40 kDa
25 kDa
15 kDa
10 kDa
5 kDa
2 kDa
L1+ L5 Total Cell Lysate
L2+ L6 Protein+(6X HisTag)
L3+ L7 Purified Protein

```
fhub        ----------MSKRIALFPALLLALLVIVA--------TALTWMNFSQALPRSQWAQAAW    42
1033-1034   MQNLIIGIQKRKNRITLFSSLFLLIIISLSFFILLIGDESYSFSTLIKVLNSETVPGA--    58
                       .:**:** :*:* ::: ::         : :: .: :.*  .    *

fhub        SPDIDVIEQMIFHYSLLPRLAISLLVGAGLGLVGVLFQQVLRNPLAEPTTLGVATGAQLG    102
1033-1034   --------SFSIMEIRLPKLLAGIIAGWSFGLAGFIFQTMLRNPLASPDIIGVTSSSSIA    110
                    .: :    **:*  .::.* .:**.*.:** :******.*  :**::::::.

fhub        ITVTTLWAIPGAMASQFAAQAGACVVGLIVFGVAWGKRLSPVTLILAGLVVSLYC-GAIN    161
1033-1034   AVFCILVLKTNSLTTGIISITCGLTSSLILFLLAKKDGFSAARLIILGIGFQAVTRAGTS    170
             ..  *    .:::: : : : . . .**:* :*  . :* . **: *: ..    .. .

fhub        QLLVIFHHDQLQSMFLWSTGTLTQTDWGGVERLWPQLLGGVMLTLLLLRPLTLMGLDDGV    221
1033-1034   FLLLKVARYELQEVMRWLSGSLSFTKLDDIPLVLIVSIIATILVLFFNKRLEIIELGEEI    230
             **: . : :**.:: * :*:*: *. ..:  :    : ..:*.*:: : * :: *.: :

fhub        ARNLGLALSLARLAALSLAIVISALLVNAVGIIGFIGLFAPLLAKMLGARRLLPRLMLAS    281
1033-1034   AIGLGANPELSRLVLIFCAVSLTAFSTSITGPIACISFLAGPIALNIGKKR---SPILAG    287
            * .**   .*:**. :  *: ::*: .. .* *. *.::*  :*  :* :*     :**.

fhub        LIGALILWLSDQIILWLTRVWMEVSTGSVTALIGAPLLLWLLPRLRSISAPDMKVNDRVA    341
1033-1034   LVGILLVLLSDIFSQN--ILPARYPVGVVTGLLGSPYLIYLLIKMNRRNIMHAKMRNKKQ    345
            *:* *:: *** :      :  .  .* **.*:*:* *::** ::.  .  . *:.::

fhub        AERQHVLAFALAGGVLLLMAVVVALSFGRD---------AHGWTWASGALLEDLMPWRWP    392
1033-1034   INL--GIIFIICLGLL--ITIFLSLKLGTKEINIRDFLAAFGMGNTNDDFIKSIIYKRIP    401
             :    : * :. *:*  :::.::*.:* .         *.* :..   :::.::  * *

fhub        RIMAALFAGVMLAVAGCIIQRLTGNPMASPEVLGISSGAAFGVVLMLFLVPGNAFGWLLP    452
1033-1034   RTIFAILAGSSLAISGVLMQSVTRNPIADPGILGINTGASLSVVIGLSFLGISSSISHIS    461
            * : *::**  **::* ::* :* **:*.* :***.:**::.**: * ::  .:    :

fhub        AGSLGAAV--TLLIIMIAAGRGGFSPHRMLLAGMALSTAFTMLLMMLQASGDPRMAQVLT    510
1033-1034   FAIIGGLVSAIFVYAIAVSGKAGLTPIKLALSGTCVSMALSSFVSFLILPNNNVLDKFRF    521
             . :*. *   ::  : .:*:.*::* :: *:* .:* *:: :: :*   .:  : :.

fhub        WISGSTYNATDAQVWRTGIVMVIL-LAITPLCRRWLTILPLGGDTARAVGMALTPTRIAL    569
1033-1034   WQIGSLGAATLSSISTL-LPFIILGHLIAIFISSDLNALAMGDEMAVGLGVNVNRIRSLA    580
            *  **   ** :.:    : ::**   *: :    *. * :*.: * .:*: :.  *

fhub        LLLAACLTATATMTIGPLSFVGLMAPHIARMMGFRRTMPHIVISALVGGLLLVFADWCGR    629
1033-1034   IIASVLLCSSITAIGGPIGFVGLIVPHFCGLFISKDIRTMTISSSFIGAELLLICDIIGR    640
            :: :. * :: *   **:.****:.**:. ::  :      : *:::*. **::.*  **

fhub        MVLFPFQIPAGLLSTFIGAPYFIYLLRKQSR---      660
1033-1034   MLGKPGEIEVGIITAIIGGPVLIYVTMKNRGVNN      674
            *:  * :* .*:::::**.* :**:  *:
```
