# Supplementary material for: Iron and its import systems enhance copper accumulation in Streptococcus pneumoniae
Source: mSphere. 2026 Jun 10;11(6):e00165-26. doi: 10.1128/msphere.00165-26 (PMC13317198; doi:10.1128/msphere.00165-26)
Supplement: Legends — Supplemental figure legends. [file msphere.00165-26-s0002.docx]

Supplemental figure legends:

Supplemental Figure 1. Common laboratory growth media contain diverse concentrations of trace metals. Bar graph showing quantified levels of trace metals using ICP-OES. Statistical significance was determined via One-way ANOVA with Tukey’s multiple comparison (ns, non-significant; *, P < 0.05; **, P <0.01; ***, P <0.001; ****, P <0.0001).

Supplemental Figure 2. Schematic of the iron-replete experiment. Parallel cultures were grown on RPMI_mod_ and incubated at 37ºC. Culture A contained 100 µM FeSO_4_ during the initial incubation and was determined as iron replete. Culture B contained only trace metal iron in the culture medium. Both cultures were grown to the exponential phase, then centrifuged and resuspended in fresh medium. Each culture was then divided into four distinct treatments: control, Cu 200 µM, Fe 200 µM and both copper and iron and incubated for an additional 30 minutes.

Supplemental Figure 3. Volcano plots displaying differential gene expression of TIGR4 grown on RPMI_mod_ under different metal exposure in comparison to control. A) Gene expression of TIGR4 exposed to 200µM iron for 30 minutes. B) Gene expression of TIGR4 exposed to 200µM copper and iron for 30 minutes. C) Gene expression of TIGR4 grown with 100 µM Iron, then exposed to 200µM copper and iron for 30 minutes.

Supplemental Figure 4. Protein counts of particular metal related proteins after 30 minutes of different metal exposures. A-B) Proteins involved in iron homeostasis. C) Proteins involved in manganese homeostasis. D) Proteins involved in zinc homeostasis.

Supplemental Figure 5. Bar graph showing CFU counts at baseline and 90 minutes after metal washout period. Statistical significance was determined with a Mann-Whitney test (ns, non-significant; *, P < 0.05; **, P <0.01; ***, P <0.001; ****, P <0.0001).

Supplemental Figure 6. Growth curves showing iron transport mutants and wild type growing on RPMI_mod_. Iron transport mutants display a longer lag phase.

Supplemental Figure 7. Iron transport mutants display similar iron-enhanced copper accumulation under Fe(III) conditions. Bar graphs showing metal content under diverse conditions. Bacteria were grown on RPMI_mod_. (A-D) Copper content. (E-H) Iron content. Statistical differences were determined using a One-way ANOVA with Tukey’s multiple comparison’s test (ns, non-significant; *, P < 0.05; **, P <0.01; ***, P <0.001; ****, P <0.0001).

Supplemental Figure 8. Metal accumulation differences in mutants and TIGR4. A-F) Bar graphs display intracellular levels of indicated trace metals after 30 minutes of indicated treatments (exposure to copper, iron, combination or no exposure). Statistical differences were determined using a One-way ANOVA with Tukey’s multiple comparison’s test (ns, non-significant; *, P < 0.05; **, P <0.01; ***, P <0.001; ****, P <0.0001).

Supplemental Figure 9. SDS PAGE gel showing the purification of iron substrate-binding proteins PiaA and PiuA.

Supplemental Figure 10. Clustal alignment of the permease Fhub in *E. coli* against PiaB,C from pneumococcus. Showing conserved amino acid residues or conserved amino acid properties.
